# Chemoenzymatic Dynamic Kinetic Resolution of Atropoisomeric 2‑(Quinolin-8-yl)benzylalcohols

**DOI:** 10.1021/acs.joc.4c02996

**Published:** 2025-04-09

**Authors:** Juan M. Coto-Cid, Valentín Hornillos, Rosario Fernández, José M. Lassaletta, Gonzalo de Gonzalo

**Affiliations:** † Facultad de Química, Departamento de Química Orgánica, Universidad de Sevilla and Centro de Innovación en Química Avanzada (ORFEO−CINQA), C/Prof. García González, 1, 41012 Sevilla, Spain; ‡ Instituto de Investigaciones Químicas (CSIC-US) and Centro de Innovación en Química Avanzada (ORFEO−CINQA), Avda. Américo Vespucio, 49, 41092 Sevilla, Spain

## Abstract

The chemoenzymatic dynamic kinetic resolution of 2-(quinolin-8-yl)­benzylalcohols using a combination of lipases and ruthenium catalysts is described. While CalB lipase performs highly selective enzymatic kinetic resolution, the combination with Shvo′s or Bäckvall’s catalysts promotes atropisomerization of the substrate via the reversible formation of configurationally labile aldehydes, thereby enabling a dynamic kinetic resolution. This synergistic approach was applied to the synthesis of a variety of heterobiaryl acetates in excellent yields and enantioselectivities.

## Graphical abstract

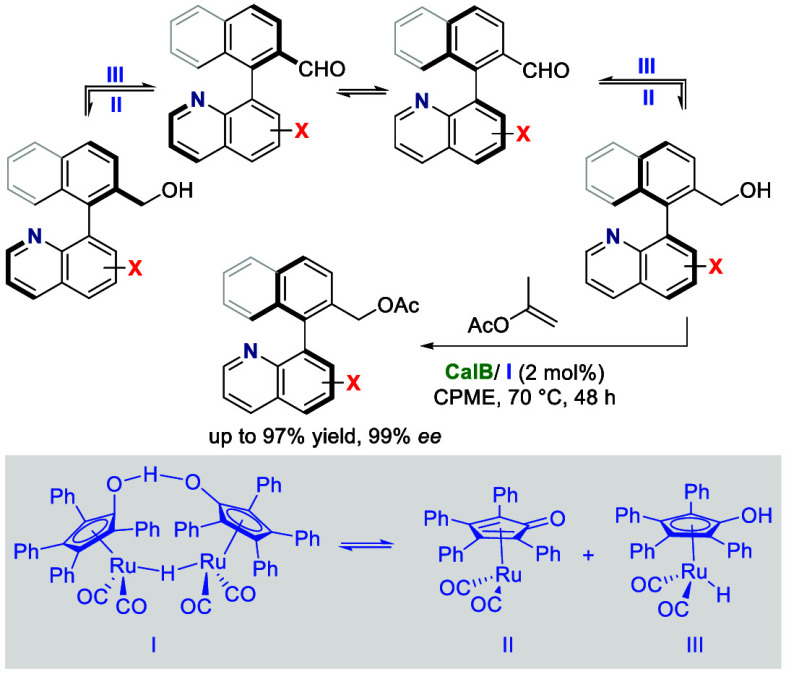

## Introduction

(Hetero)­biaryl atropisomers, particularly those containing nitrogen atoms, are essential structural elements in many natural products, biologically active compounds, and chiral ligands and catalysts.^1^ Among them, 8-arylquinoline derivatives are particularly noteworthy for their applications across these fields.^2^ Therefore, extensive efforts have been made to synthesize these axially chiral compounds using different designed strategies, which include kinetic resolution (KR) by asymmetric transfer hydrogenation,^3^ atroposelective halogenation,^4^ cross-coupling reaction,^5^ and enantioselective C–H olefinations.^6^

On the other hand, we recently reported on a dynamic kinetic resolution (DKR) strategy that exploits transient Lewis acid–Lewis base interactions^7^ for the dynamization of (hetero)­biaryl carbonyl compounds strategically functionalized with basic nitrogen-,^8^ sulfur-,^9^ or phosphorus-based groups.^10^ In these systems, catalytic reactions that result in the quaternization of the carbonyl compound provide easy access to a variety of functionalized axially chiral derivatives (Scheme 1).

**Scheme 1:**
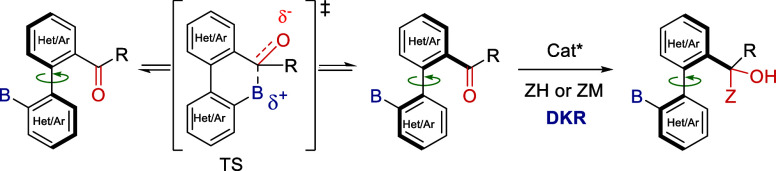
Dynamization Strategy via Transient Lewis Acid–Lewis Base Interactions

This strategy has been applied to the synthesis of a series of quinoline-containing biaryl alcohols from their corresponding aldehydes, utilizing both Ir-catalyzed asymmetric allylation^8^ and biocatalytic reductions via alcohol dehydrogenases (ADHs).^11^ Notably, the biocatalytic approach yielded both atropoisomers of the final products with high yields and enantioselectivities through the careful selection of the biocatalyst and substrate structure. However, the use of isolated ADHs in asymmetric synthesis may encounter challenges when scaling up, as it requires low substrate concentrations and necessitates the implementation of nicotinamide cofactor recycling systems. Conversely, lipases are robust and versatile enzymes that perform well in organic solvents, making them suitable for a wide range of synthetic procedures.^12^ These biocatalysts have been extensively employed in the KR of heterobiaryl compounds through acylation, allowing the recovery of enantiopure starting materials and final esters with high enantioselectivities, albeit limited to the maximum theoretical 50% yield of the KR process.^13^ To resolve this issue, DKR methods based on the combination of lipases and an additional racemization catalyst have been developed for the resolution of secondary alcohols (Scheme 2A).^14^

**Scheme 2:**
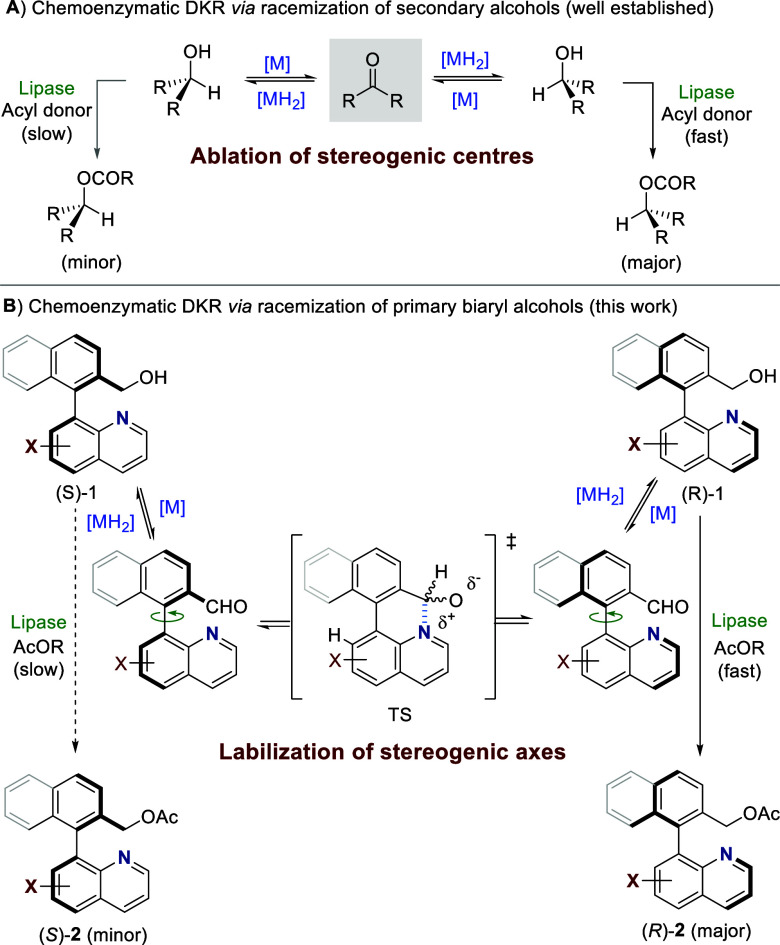
Chemoenzymatic Dynamic Resolutions with Lipases

On this basis, we envisaged that the combination of lipases with a transition metal catalyst could also be applied to the chemoenzymatic DKR of primary biaryl alcohols **1**, exploiting in this case the configurational lability of the aldehyde **2** (generated in situ by dehydrogenation) facilitated by the above-mentioned Lewis acid–Lewis base interaction in the transition state for the atropisomerization (Scheme 2B).

It should be noticed that there are only two reports dealing with the chemoenzymatic generation of axial chirality via DKR, namely, the resolution of axially chiral allenes by Bäckvall and co-workers using a Pd-catalyzed allene racemization^15^ and the biocatalytic DKR of BINOL derivatives reported by Akai and co-workers, involving a Ru­(II)-catalyzed racemization proceeding via free radicals.^16^ However, none of these processes rely on the hydrogen-borrowing racemization mechanism commonly used in the chemoenzymatic synthesis of chiral carbinols.

## Results and Discussion

As the first task, we started experiments to identify the optimal catalyst and conditions for the KR of racemic (1-(quinolin-8-yl)­naphthalen-2-yl)­methanol (±)-**1a**, synthesized as previously described,^10^ as a model substrate in the presence of vinyl acetate as the acyl donor. Different commercially available lipases (Sigma-Aldrich) were studied for KRs performed in toluene at 30 °C. No reaction was observed in the presence of Pseudomonas sp. lipase (PSL), Candida rugosa lipase (CRL), or porcine pancreas lipase (PPL), whereas the isoenzyme A from Candida antarctica (CalA) leads to a resolution process with 44% conversion albeit in an unselective way.^17^ A significant improvement was observed in the Candida antarctica B (CalB)-catalyzed acetylation, which afforded 38% (*R*)-**2a** after 24 h with high enantioselectivity (*E* = 120, Table 1, entry 1).^18^ Using this enzyme, different parameters were then analyzed. Initially, a range of organic solvents with varying properties were examined at 30 °C.^17^ Significant selectivity factors were achieved using diethyl ether (entry 2) and vinyl acetate, employed as both the solvent and acyl donor (entry 3). Diethyl ether exhibited a high *E* value (*E* = 96), leading to a 41% conversion, whereas vinyl acetate showed a slightly lower enantioselectivity. The most favorable outcomes for this process, aside from using toluene, were achieved by selecting methyl *tert*-butyl ether (MTBE) or cyclopentyl methyl ether (CPME), with *E* > 100 and ∼50% conversions after 24 h (entries 4 and 5). Among these three solvents, CPME was selected for its high boiling point, conduciveness to further dynamic processes, and its classification as a biobased solvent derived from renewable sources, contributing to a more sustainable catalytic procedure.^19^

**Table 1:**
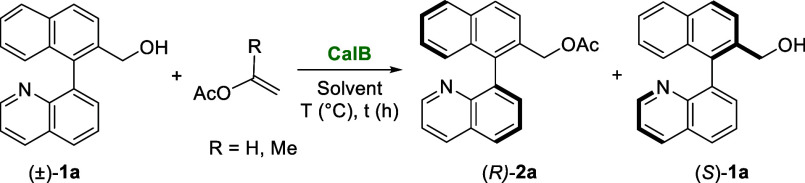
KR of Racemic Alcohol (±)-**1a** (0.1 mmol) Employing Lipases in Acetylation Reactions^a^

entry	solvent	*T* (°C)/*t* (h)	*c* (%)^b^	ee **1a** (%)^c^	ee **2a** (%)^c^	*E* ^d^
1	Toluene	30/24	38	59	97	120
2	Et_2_O	30/30	20	41	67	96
3	Vinyl acetate	30/30	2	26	34	97
4	MTBE	30/20	49	92	96	168
5	CPME	30/20	50	94	95	139
6	CPME	45/3	34	52	99	>200
7	CPME	60/3	42	69	99	>200
8	CPME	70/1.5	45	81	98	>200
9	Toluene	70/3	45	78	97	160
10^e^	CPME	45/3	34	52	99	>200
11^e^	CPME	70/2.5	48	90	97	>200
12^e^	Toluene	70/2.5	30	40	98	146

a Reactions conditions: 50 mM **1a**.

b Conversions were determined from the enantiomeric excesses, *c* = ee**_1a_**/ (ee**_1a_** + ee**_2a_**).

c Enantiomeric excesses were determined by HPLC on chiral stationary phases.

d Enantioselectivity or enantiomeric ratio, *E* = ln­[(1 – ee_s_)­(1 + ee_s_/ee_p_)]/ ln­[(1 + ee_s_)­(1 + ee_s_/ee_p_)].

e Isopropenyl acetate (R = Me) was employed as the acyl donor instead of vinyl acetate (R = H).

Since racemization catalysts typically require higher temperatures for optimal performance, the resolution of (±)-**1a** was conducted at 45, 60, and 70 °C. High enantioselectivities and excellent conversions were observed at low reaction times for all tested temperatures (entries 6, 7, and 8), highlighting the high thermostability of CalB. Toluene was also used as a solvent in the CalB-catalyzed acylation at 70 °C, resulting in a 45% yield of (*R*)-**2a** with a high selectivity factor (*E* = 160, entry 9). Finally, the use of isopropenyl acetate as an acyl donor in reactions catalyzed by CalB in CPME at 45 and 70 °C, as well as in toluene at 70 °C, resulted in excellent enantioselectivities, with slightly lower conversions compared to those obtained in the presence of vinyl acetate (Table 1, entries 10, 11, and 12).

As CalB was employed as an immobilized catalyst, its recycling across various temperatures was performed (see Table S3). At 45 °C, CalB retained its activity and selectivity for four cycles, but a decrease in selectivity was observed by the fifth cycle. At 70 °C, the stability of the biocatalyst is compromised, leading to a decrease in enantioselectivity starting from the second cycle. However, high enantioselectivity values (*E* values around 100) can still be achieved up to the fourth cycle.

The optimal conditions identified for the KR of (±)-**1a** using CalB and vinyl acetate in CPME were successfully applied for the resolution of different substituted heterobiaryl alcohols presenting substituents at the 5- or 6- position of the quinoline moiety (**1b**–**e**). Excellent results were achieved both at 45 °C (see Table S4, Supporting Information) and 70 °C (Scheme 3). Except for the 6-methyl derivative (**1e**), which exhibits good enantioselectivity but moderate conversion (17% (*R*)-**2e** after 3 h, *E* = 80), all other racemic substrates were effectively resolved using CalB. Thus, the 5-trifluoromethyl (**1b**), 5-chloro (**1c**), and 6-fluoro (**1d**) alcohols were acetylated, with *E* values exceeding 100, in good conversion (28–38%) after 3 h (Scheme 3). The starting material structure was further modified by replacing the naphthyl group on the aromatic ring with a tolyl group. The KR of racemic (3-methyl-2-(quinolin-8-yl)­phenyl)­methanol (±)-**1f** resulted in 49% conversion with excellent selectivity. 6-Methyl (**1g**), 5-fluoro (**1h**), and 5-chloro (**1i**) tolyl alcohols were also acetylated with conversions around 40% in short times and with excellent enantioselectivities (*E* values higher than 149).

**Scheme 3:**
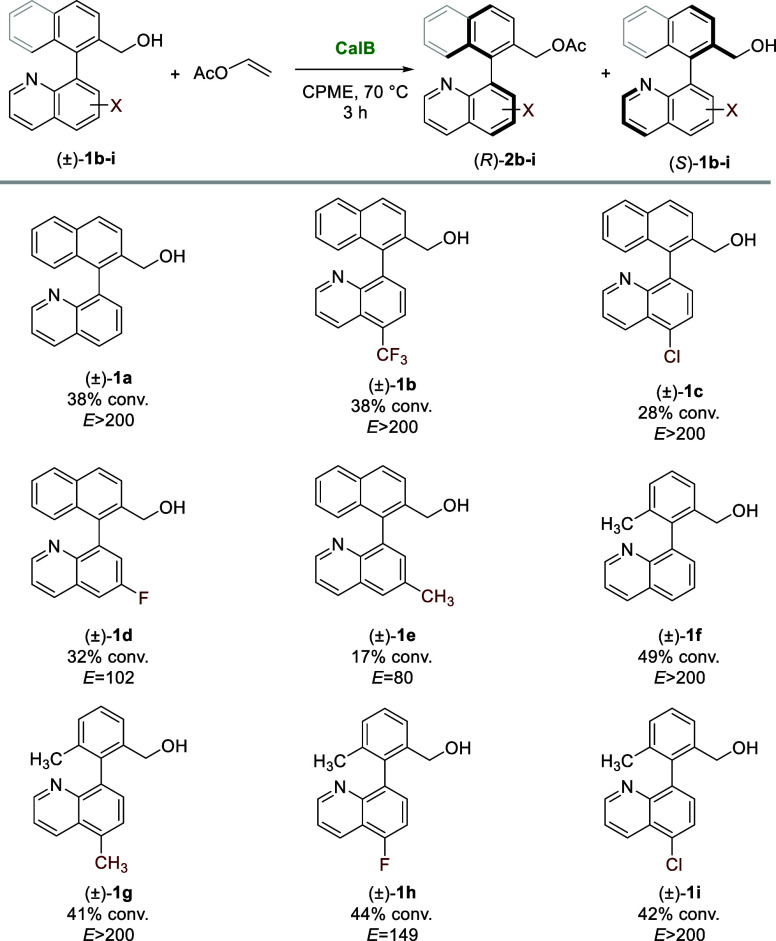
KR of Racemic Heterobiaryl Alcohols (±)-**1a**–**i** (0.05 mmol) Catalyzed by CalB (15 mg) in CPME (2 mL) in the Presence of Vinyl Acetate (0.15 mmol) as the Acyl Donor

After establishing that the racemic aryl quinolyl alcohols (±)-**1a**–**i** undergo highly selective KR via acylation using CalB, the development of DKR procedures was undertaken. This involved combining the established biocatalyzed KR with substrate racemization through an oxidation–reduction of the primary alcohol using a metal catalyst. Although several examples of DKR procedures for secondary alcohols using this methodology have been described,^14^ very few primary racemic alcohols have been tested in these processes, none of them leading to aromatic aldehydes.^15,20^ Several metal catalysts were applied in the DKR of substrate (±)-**1a** in the presence of CalB using either vinyl or isopropenyl acetate (see Supporting Information). When using vinyl acetate, the Shvo′s catalyst (**I**)^21^ (2 mol %) afforded (*R*)-**2a** in 94% conversion and 80% ee (see entry 2, Table S5), also achieving a notable effectiveness in the presence of the Bäckvall’s catalyst, thus recovering the (*R*)-ester in 78 and 84% ee. Shvo′s catalyst yielded the best result in the DKR employing CPME with isopropenyl acetate as the acyl donor, leading to chiral acetate (*R*)-**2a** in 97 and 98% ee after 24 h at 70 °C, as shown in Scheme 4. ^1^H NMR analysis confirmed the absence of starting material **1a**, with the remaining 3% of the reaction comprising aldehyde **3a**, which was not completely reduced in the redox racemization process.

**Scheme 4:**
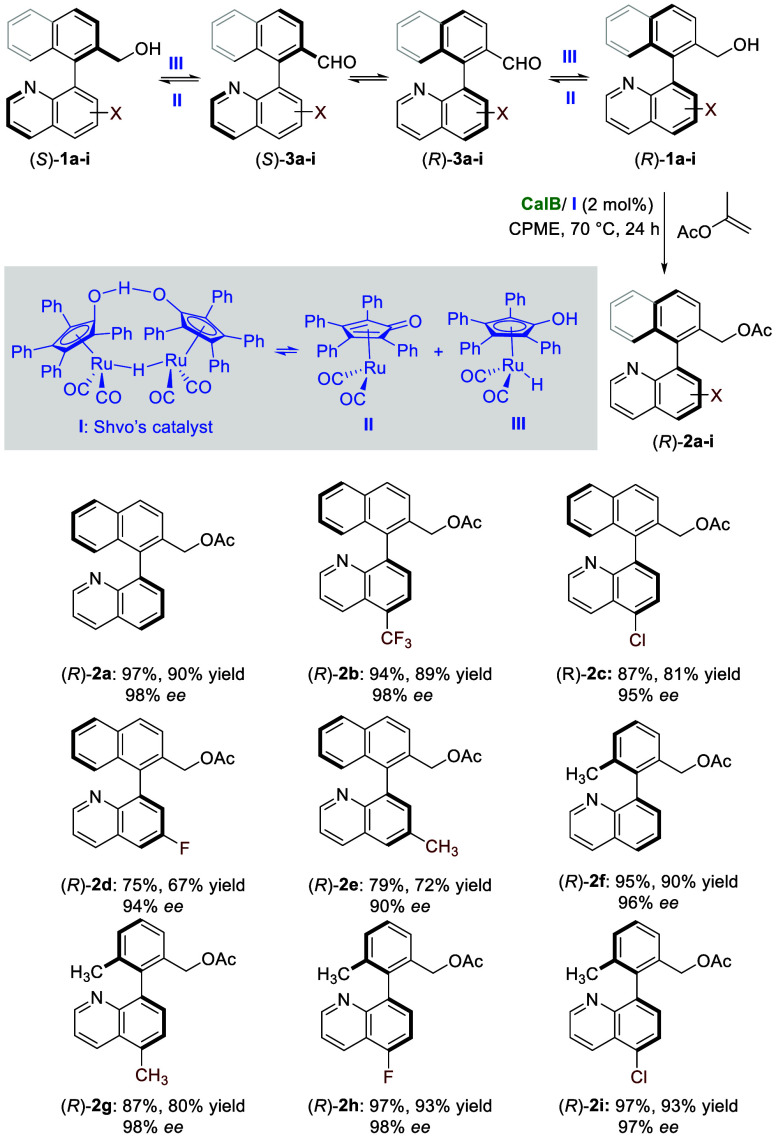
Chemoenzymatic DKR of Racemic Heterobiaryl Alcohols (±)-**1a**–**i** (0.1 mmol) using CalB (30 mg) and Shvo’s Catalyst (2 mol %)

The DKR in these optimized conditions of other naphthyl quinoline alcohol-containing substituents on the quinolyl ring afforded (*R*)-acetates **2b**–**e** in amounts ranging from 75 to 94% and optical purities higher than 90% (Scheme 4). No starting alcohol **1b**–**e** was detected for these compounds, with 6–25% of the corresponding aldehydes **3b**–**e** recovered. The DKR of the tolyl quinoline derivatives yielded better results, with the (*R*)-esters being recovered in excellent enantiomeric excesses (96–98%) and high percentages (87–97%). No alcohol was observed, with aldehydes **3f**–**i** being achieved as the sole byproducts of the dynamic process.

To demonstrate the applicability of this procedure, a scale-up of the DKR of (±)-**1a** was developed employing 2.10 mmol of this substrate (600 mg) in CPME (40 mL) using CalB (500 mg) and catalyst **I** (45.6 mg, 2 mol %), as described in the Supporting Information. After 24 h and flash chromatography purification, 606.0 mg of isolated (*R*)-**2a** (97% ee) were recovered with 88% yield,

## Conclusions

The lipase B from Candida antarctica can be effectively employed in the KR of a set of 2-(quinolin-8- yl)­benzylalcohols by selective acetylation. High conversions and enantioselectivities were achieved when working in toluene or cyclopentyl methyl ether at 70 °C, yielding the corresponding (*S*)-alcohols and (*R*)-esters with excellent enantioselectivities and conversions nearing 50%. The combined use of the Shvo’s ruthenium catalyst for the substrate racemization with the enantioselective lipase-catalyzed KR enables the efficient synthesis of a set of (*R*)-esters with high yields and excellent optical purities from readily accessible racemic substrates by DKR. This method represents the first instance where the biocatalyzed acylation of axially chiral primary alcohols has been integrated with metal-catalyzed substrate atropisomerization to achieve an effective DKR process.

## Supplementary Material



## Data Availability

The data underlying this study are available in the published article and its Supporting Information.

## Notes

Experimental details, optimizations, spectroscopic and analytical data for new compounds, and HPLC spectra of alcohols **1a**–**i** and esters **2a**–**i** (PDF)

## Notes

Dedicated to the memory of Prof. Iván Lavandera.
