# Supplementary material for: Chemoenzymatic Dynamic Kinetic Resolution of Atropoisomeric 2‑(Quinolin-8-yl)benzylalcohols
Source: J Org Chem. 2025 Apr 9;90(15):5120–4. doi: 10.1021/acs.joc.4c02996 (PMC12160057; doi:10.1021/acs.joc.4c02996)
Supplement: Supplementary file 1 [file jo4c02996_si_001.pdf]

Chemoenzymatic Dynamic Kinetic Resolution of Atropoisomeric 2-(Quinolin-8-yl)benzylalcohols

Juan M. Coto-Cid,† Valentin Hornillos,†,* Rosario Fernández,† José M. Lassaletta,§,* and Gonzalo de Gonzalo†,*

† Departamento de Química Orgánica, Universidad de Sevilla and Centro de Innovación en Química Avanzada (ORFEO-CINQA), C/ Prof. García González, 1, 41012 Sevilla, Spain. E-mail: vhornillos@us.es; gdegonzalo@us.es.

§ Instituto de Investigaciones Químicas and Centro de Innovación en Química Avanzada (ORFEO-CINQA), Avda/ Américo Vespucio 49, 41092 Sevilla, Spain. E-mail: jmlassa@iiq.csic.es

Index

1. General information...S3

2. Experimental Section...S4

2.1. General procedure for the chemical acetylation of 1-(quinolin-8-yl)-napthalen-2-yl and 3-methyl-2-(quinolin-8-yl)phenyl alcohols **1a-i**..S4

2.2. Lipase-catalyzed acylation of heterobiaryl alcohol (±)-**1a**...................................S7

2.2.1. Optimization of the reaction parameters in the kinetic resolution of alcohol (±)-**1a**...S7

2.2.2. CalB-recycling at the kinetic resolution of (±)-**1a**..S8

2.3. General procedure for the kinetic resolution of heterobiaryl alcohols (±)-**1b-i**..............S9

2.4. General procedure for the Dynamic Kinetic Resolution of racemic heterobiaryl alcohols **1a-i** in presence of CalB and metal catalysts (**I-IX**)..S12

2.4.1. DKRs of alcohols (±)-**1a-i** employing CalB and 1-hydroxytetraphenyl-cyclopentadienyl(tetraphenyl-2,4-cyclopentadien-1-one)-μ-hydrotetracarbonyldiruthenium(II) (Shvo's catalyst, **I**)..S12

2.4.2. DKR or racemic alcohol (±)-**1a** in presence of lipase CalB and chlorodicarbonyl(1,2,3,4,5-pentaphenylcyclopentadienyl)ruthenium(II) (Bäckvall´s catalyst, **II**)...S13

2.4.3. DKR of (±)-**1a** employing CalB and chloro(indenyl)bis(triphenylphosphine)ruthenium (**III**)...S14

2.4.4. DKR of (±)-**1a** in presence of CalB and chloro(pentamethylcyclopentadienyl)(cyclooctadiene)ruthenium(II) (**IV**)......................S14

2.4.5. DKR of (±)-**1a** employing CalB and pentamethylcyclopentadienyliridium(III) chloride dimer (**V**)...S15

2.4.6. DKR of (±)-**1a** in presence of CalB and benzeneruthenium(II) chloride dimer (**VI**) or dichloro(1,5-cyclooctadiene)ruthenium(II) polymer (**VII**)......................................S15

2.4.7. DKR of (±)-**1a** in presence of CalB and trimethylaluminum (**VIII**).....................S16

2.4.8. DKR of (±)-**1a** in presence of CalB and vanadium(IV) oxide sulfate hydrate (**IX**)....S16

2.5. DKR of (±)-**1a** employing CalB and Shvo's catalyst at high scale..........................S16

3. References...S18

4. NMR and HPLC spectra of compounds **2a-i**..S19

1. General information

Nuclear magnetic resonance (NMR) spectra were obtained at different frequencies: 500 MHz for ^{1}H-NMR, 125 MHz for ^{13}C-NMR and 471 MHz for ^{19}F-NMR. The solvent peak served as the internal reference, with chemical shifts of 7.26 ppm for ^{1}H and 77.0 ppm for ^{13}C, using $CDCl_3$ as the solvent. Column chromatography was conducted on silica gel (Merck Kieselgel 60), and analytical thin-layer chromatography (TLC) was performed on aluminum-backed plates (1.5 × 5.0 cm) coated with 0.25 mm of silica gel (Merck, Silica Gel 60 F254). Compounds were visualized either by exposing the plates to UV light or by immersing them in a solution containing 5% $(NH_4)_2Mo_7O_{24}\cdot 4\ H_2O$ in 95% EtOH (w/v) followed by heating. Reactions were continuously monitored using TLC and/or NMR analysis. The final products were characterized using ^{1}H-NMR, ^{13}C-NMR, and high-resolution mass spectrometry. Optical rotations were determined using a JASCO P-2000 polarimeter. High-performance liquid chromatography (HPLC) analyses were performed with a Thermo-Fisher UltiMate chromatograph equipped with a Thermo UltiMate detector. Unless otherwise specified, analytical-grade solvents and commercially available reagents were used without further purification. Starting alcohols **1a-l** were synthesized according to the literature.[1] Lipases from *Candida antarctica* A (CalA, ≥500 units/g), *Candida antarctica* B (CalB, ≥1800 units/g), *Candida rugosa* (CRL, ≥700 units/mg solid), *Burkholderia cepacia* (PSL, ≥30,000 units/g) and porcine pancreas (PPL, ≥125 units/mg protein) were products from Sigma-Aldrich. Shvo’s (**I**) and Bäckvall’s (**II**) catalysts, as well as **IV**, **V**, **VIII** and **IX** were products from Sigma-Aldrich. Catalysts **III**, **VI** and **VII**, as well as ligands $\mathbf{L_1}$ and $\mathbf{L_3}$ were purchased from BLDPharm. Ligands $\mathbf{L_2}$ and $\mathbf{L_4}$ were obtained from TCI Europe. All other reagents and solvents were from commercial sources. Absolute configuration of chiral esters (*R*)-**2a-i** has been previously established.[2]

2. Experimental Section

2.1. General procedure for the chemical acetylation of 1-(quinolin-8-yl)-napthalen-2-yl and 3-methyl-2-(quinolin-8-yl)phenyl alcohols 1a-i.

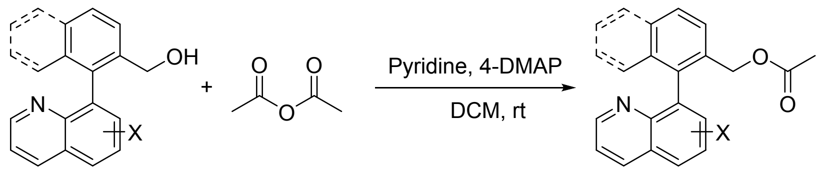

To a solution of the corresponding racemic alcohol (±)-**1a-i** (0.3 mmol) and pyridine (0.6 mmol) in andydrous dicloromethane (DCM, 2.0 mL), acetic anhydride (0.6 mmol) and a catalytic amount of 4-dimethylaminopyridine (4-DMAP) were added. The mixture was stirred at room temperature, being monitored by TLC in using *n*-hexane:EtOAc (1:1) as eluent. Once finished, the crude was poured employing brine (2.0 mL) followed by the extraction with DCM (2 x 5 mL). The organic layers were combined, dried onto $MgSO_4$ and concentrated under reduced pressure. Further purification was carried out by flash chromatography using *n*-hexane:EtOAc (1:1) as eluent, to obtain the corresponding racemic esters (±)-**2a-i**.

(±)-(1-(quinolin-8-yl)naphthalen-2-yl)methyl acetate (2a). Following the general procedure,

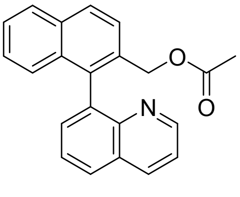

(±)-**2a** was obtained as a yellow pale oil (95.2 mg, 97% yield). **^{1}H NMR** (500 MHz, Chloroform-d) δ 8.79 (dd, *J* = 4.2, 1.8 Hz, 1H), 8.26 (dd, *J* = 8.4, 1.8 Hz, 1H), 7.97 (d, J = 7.9 Hz, 2H), 7.91 (d, J = 8.2 Hz, 1H), 7.72 – 7.63 (m, 3H), 7.44 (ddd, *J* = 8.1, 6.7, 1.2 Hz, 1H), 7.39 (dd, *J* = 8.3, 4.1 Hz, 1H), 7.29 – 7.22 (m, 1H), 7.18 (d, J = 8.5 Hz, 1H), 4.90 (dd, J = 83.6, 12.6 Hz, 2H), 1.91 (s, 3H). **^{13}C NMR{^{1}H}** (100 MHz, Chloroform-d) δ 170.8, 150.8, 147.5, 137.4, 137.2, 136.4, 133.3, 133.2, 131.9, 131.8, 128.6, 128.5, 128.4, 128.1, 126.8, 126.2, 126.2, 126.0, 121.4, 65.3, 20.9. **HRMS (ESI)** calcd. for $C_{22}H_{18}NO_2$ (M + H^+) 328.1332. Found 328.1337.

(±)-(1-(5-(trifluoromethyl)quinolin-8-yl)naphthalen-2-yl)methyl acetate (2b). Following

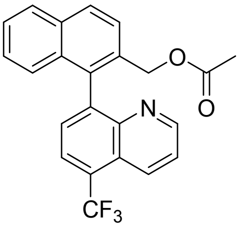

the general procedure, (±)-**2b** was obtained as a yellow pale oil (106.6 mg, 90% yield). **^{1}H NMR** (500 MHz, Chloroform-*d*) δ 8.84 (d, *J* = 4.1 Hz, 1H), 8.62 (d, *J* = 8.7 Hz, 1H), 8.07 (d, *J* = 7.5 Hz, 1H), 8.00 (d, *J* = 8.5 Hz, 1H), 7.92 (d, *J* = 8.2 Hz, 1H), 7.71 (dd, *J* = 23.4, 8.0 Hz, 2H), 7.53 (dd, *J* = 8.7, 4.1 Hz, 1H), 7.49 – 7.43 (m, 1H), 7.29 – 7.24 (m, 1H), 7.08 (d, *J* = 8.5 Hz, 1H), 4.87 (dd, *J* = 97.3, 12.7 Hz, 2H), 1.92 (s, 3H). **^{13}C NMR{^{1}H}** (125 MHz, Chloroform-*d*) δ 170.8, 151.3, 147.7, 142.7, 136.1, 133.3, 132.9, 131.8, 130.4, 129.9, 128.8, 128.2, 126.8 (d, *J* = 30.8 Hz), 126.3 (d, *J* = 35.6 Hz), 124.3 (d, *J* = 273.7 Hz), 122.5, 65.0, 20.9.

^{19}F NMR (471 MHz, Chloroform-*d*) δ -58.99. **HRMS (ESI)** calcd. for $C_{23}H_{17}F_3NO_2$ (M + H$^+$) 396.1206. Found 396.1205.

(±)-(1-(5-chloroquinolin-8-yl)naphthalen-2-yl)methyl acetate (2c). Following the general procedure, (±)-**2c** was obtained as a yellow pale oil (104.0 mg, 96% yield). **^{1}H NMR** (500 MHz, Chloroform-*d*) δ 8.81 (dd, *J* = 4.1, 1.8 Hz, 1H), 8.70 (dd, *J* = 8.5, 1.7 Hz, 1H), 7.98 (d, *J* = 8.5 Hz, 1H), 7.91 (d, *J* = 8.2 Hz, 1H), 7.78 (d, *J* = 7.7 Hz, 1H), 7.68 (d, *J* = 8.6 Hz, 1H), 7.60 (d, *J* = 7.7 Hz, 1H), 7.50 (dd, *J* = 8.5, 4.1 Hz, 1H), 7.45 (m, 1H), 7.26 (dd, *J* = 15.3, 1.3 Hz, 1H), 7.15 (d, *J* = 8.5 Hz, 1H), 4.88 (dd, *J* = 85.0, 12.6 Hz, 2H), 1.93 (s, 3H). **^{13}C NMR{^{1}H}** (125 MHz, Chloroform-*d*) δ 170.8, 151.3, 148.0, 133.3, 133.2, 133.2, 132.0, 131.7, 131.6, 128.6, 128.2, 126.7, 126.6, 126.4, 126.3, 126.2, 126.1, 122.1, 65.1, 20.9. HRMS (ESI) calcd. for $C_{22}H_{17}ClNO_2$ (M + H$^+$) 362.0942. Found 362.0945.

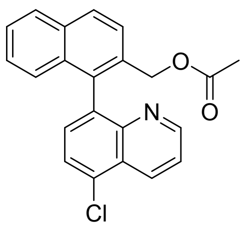

(±)-(1-(6-fluoroquinolin-8-yl)naphthalen-2-yl)methyl acetate (2d). Following the general procedure, (±)-**2d** was obtained as a yellow pale oil (98.3 mg, 95% yield) **^{1}H NMR** (500 MHz, Chloroform-*d*) δ 8.73 (dd, *J* = 4.1, 1.7 Hz, 1H), 8.21 (dd, *J* = 8.4, 1.8 Hz, 1H), 7.98 (d, *J* = 8.5 Hz, 1H), 7.91 (d, *J* = 8.2 Hz, 1H), 7.68 (d, *J* = 8.5 Hz, 1H), 7.58 (dd, *J* = 8.5, 2.9 Hz, 1H), 7.50 – 7.43 (m, 2H), 7.41 (dd, *J* = 8.3, 4.1 Hz, 2H), 7.15 (d, *J* = 8.5 Hz, 1H), 4.88 (dd, *J* = 95.3, 12.5 Hz, 2H), 1.93 (s, 3H). **^{13}C {^{1}H}NMR** (125 MHz, Chloroform-*d*) δ 170.9, 159.8 (d, *J* = 249.2 Hz), 150.1 (d, *J* = 2.6 Hz), 144.7, 140.5 (d, *J* = 9.1 Hz), 135.9 (d, *J* = 5.6 Hz), 133.4, 133.0, 131.9, 129.4 (d, *J* = 10.2 Hz), 128.8, 128.2, 126.5 (d, *J* = 6.2 Hz), 126.4, 126.2, 122.2, 122.1, 122.0, 111.3 (d, *J* = 21.1 Hz), 65.1, 20.9. **^{19}F NMR** (471 MHz, Chloroform-*d*) δ -113.50. HRMS (ESI) calcd. for $C_{22}H_{17}FNO_2$ (M + H$^+$) 346.1238. Found 346.1239.

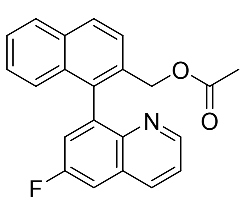

(±)-(1-(6-methylquinolin-8-yl)naphthalen-2-yl)methyl acetate (2e). Following the general procedure, (±)-**2e** was obtained as a yellow pale oil (95.1 mg, 93% yield) **^{1}H NMR** (500 MHz, Chloroform-*d*) δ 8.70 (dd, *J* = 4.1, 1.8 Hz, 1H), 8.16 (dd, *J* = 8.3, 1.8 Hz, 1H), 7.95 (d, *J* = 8.5 Hz, 1H), 7.89 (d, *J* = 8.2 Hz, 1H), 7.72 (s, 1H), 7.67 (d, *J* = 8.5 Hz, 1H), 7.49 (d, *J* = 2.0 Hz, 1H), 7.46 – 7.41 (m, 1H), 7.34 (dd, *J* = 8.3, 4.1 Hz, 1H), 7.26 (d, *J* = 4.4 Hz, 1H), 7.18 (dd, *J* = 8.5, 1.2 Hz, 1H), 4.89 (dd, *J* = 71.9, 12.6 Hz, 2H), 2.59 (s, 3H), 1.92 (s, 3H). **^{13}C NMR{^{1}H}** (125 MHz, Chloroform-*d*) δ 170.8, 150.0, 146.01, 137.2 (d, *J* = 15.7 Hz), 135.8, 134.1, 133.2 (d, *J* = 10.2 Hz), 131.8, 131.1, 129.9, 128.7, 128.2 (d, *J* = 24.3 Hz), 127.4, 126.9, 126.2 (d, *J* = 10.9 Hz), 125.9, 121.3, 65.3, 21.7, 20.9. HRMS (ESI) calcd. for $C_{23}H_{20}NO_2$ (M + H$^+$) 342.1489. Found 342.1482.

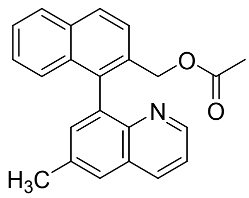

(±)-3-methyl-2-(quinolin-8-yl)benzyl acetate (2f). Following the general procedure, (±)-**2f** was obtained as a yellow pale oil (79.4 mg, 91% yield). **^{1}H NMR** (500 MHz, Chloroform-*d*) δ 8.86 (dd, *J* = 4.1, 1.8 Hz, 1H), 8.21 (dd, *J* = 8.3, 1.8 Hz, 1H), 7.87 (dd, *J* = 8.1, 1.6 Hz, 1H), 7.63 – 7.58 (m, 1H), 7.54 (dd, *J* = 7.0, 1.6 Hz, 1H), 7.41 – 7.38 (m, 1H), 7.38 – 7.35 (m, 2H), 7.32 (dd, *J* = 6.7, 2.5 Hz, 1H), 4.71 (dd, *J* = 84.9, 12.5 Hz, 2H), 1.93 (s, 3H), 1.79 (s, 3H). **^{13}C NMR{^{1}H}** (125 MHz, Chloroform-*d*) δ 170.6, 150.7, 146.7, 139.5, 138.7, 137.5, 136.4, 134.4, 130.7, 129.9, 128.7, 128.1, 127.9, 121.2, 65.3, 20.8, 20.6. HRMS (ESI) calcd. for $C_{19}H_{18}NO_2$ (M + H^+) 292.1332. Found 292.1334.

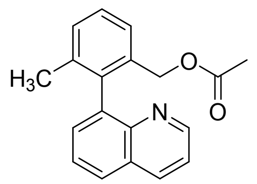

(±)-3-methyl-2-(quinolin-8-yl)benzyl acetate (2g). Following the general procedure, (±)-**1a** was obtained as a yellow pale oil (86.9 mg, 95% yield). **^{1}H NMR** (500 MHz, Chloroform-*d*) δ 8.69 (dd, *J* = 4.1, 1.5 Hz, 1H), 8.21 (dd, *J* = 8.5, 1.6 Hz, 1H), 7.29 – 7.23 (m, 3H), 7.22 – 7.18 (m, 2H), 7.16 (t, *J* = 7.9 Hz, 1H), 4.54 (dd, *J* = 66.5, 12.5 Hz, 2H), 2.59 (s, 3H), 1.76 (s, 3H), 1.66 (s, 3H). **^{13}C NMR{^{1}H}** (125 MHz, Chloroform-*d*) δ 170.7, 150.1, 146.9, 139.8, 137.6, 136.7, 134.8, 134.5, 132.7, 130.3, 129.8, 128.0, 127.8, 126.8, 126.2, 120.7, 65.3, 20.9, 20.6. HRMS (ESI) calcd. for $C_{20}H_{20}NO_2$ (M + H^+) 306.1489. Found 306.1494.

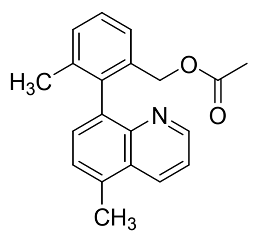

(±)-2-(5-fluoroquinolin-8-yl)-3-methylbenzyl acetate (2h). Following the general procedure, (±)-**2h** was obtained as a yellow pale oil (89.0 mg, 96% yield). **^{1}H NMR** (500 MHz, Chloroform-*d*) δ 8.78 (dd, *J* = 4.2, 1.8 Hz, 1H), 8.37 (dd, *J* = 8.4, 1.8 Hz, 1H), 7.39 – 7.31 (m, 2H), 7.28 – 7.23 (m, 2H), 7.21 – 7.19 (m, 2H), 7.18 – 7.14 (m, 1H), 4.58 (dd, *J* = 71.4, 12.5 Hz, 2H), 1.80 (s, 3H), 1.70 (s, 3H). **^{13}C NMR{^{1}H}** (125 MHz, Chloroform-*d*) δ 170.6, 157.6 (d, *J* = 255.6 Hz), 151.4, 147.1 (d, *J* = 2.9 Hz), 138.8, 137.7, 134.6 (d, *J* = 4.5 Hz), 130.0 (d, *J* = 8.6 Hz), 129.5 (d, *J* = 4.8 Hz), 128.1, 126.5, 121.3 (d, *J* = 3.0 Hz), 119.4 (d, *J* = 16.1 Hz), 109.9 (d, *J* = 19.1 Hz), 65.2, 20.8, 20.5. **^{19}F NMR** (471 MHz, Chloroform-*d*) δ -122.9. HRMS (ESI) calcd. for $C_{19}H_{17}FNO_2$ (M + H^+) 310.1238. Found 310.1243.

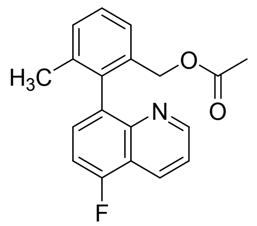

(±)-2-(5-chloroquinolin-8-yl)-3-methylbenzyl acetate (2i). Following the general procedure, (±)-**2i** was obtained as a yellow pale oil (86.8 mg, 89% yield). **^{1}H NMR** (500 MHz, Chloroform-*d*) δ 8.78 (dd, *J* = 4.1, 1.5 Hz, 1H), 8.52 (dd, *J* = 8.5, 1.5 Hz, 1H), 7.58 (d, *J* = 7.7 Hz, 1H), 7.38 (dd, *J* = 8.5, 4.1 Hz, 1H), 7.35 (d, *J* = 7.7 Hz, 1H), 7.27 – 7.23 (m, 2H), 7.20 (dd, *J* = 6.0, 2.8 Hz, 1H), 4.57 (dd, *J* = 73.9, 12.6 Hz, 2H), 1.79 (s, 3H), 1.70 (s, 3H). **^{13}C NMR{^{1}H}** (125 MHz, Chloroform-*d*) δ 170.6, 151.2, 147.3, 138.7, 138.0, 137.4, 134.4, 133.2,

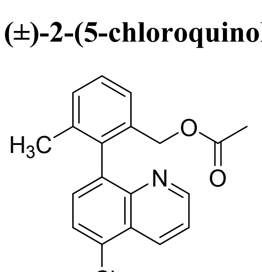

131.3, 130.4, 130.0, 128.2, 126.7, 126.4 (d, J = 3.0 Hz), 122.0, 65.1, 27.1, 20.6. HRMS (ESI) calcd. for $C_{19}H_{17}ClNO_2$ (M + H^+) 326.0942. Found 326.0946.

2.2. Lipase-catalyzed acylation of heterobiaryl alcohol (±)-1a.

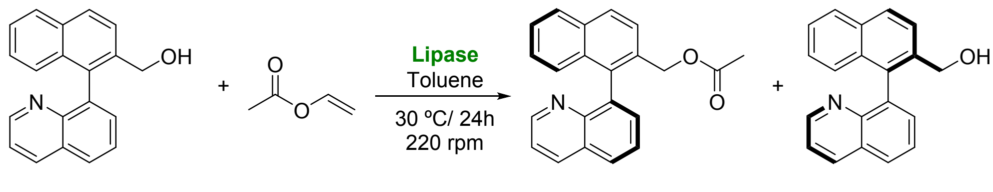

Vinyl acetate (29 µL, 0.3 mmol, 3.0 eq.) was added to a mixture of the enzyme (30 mg) and the alcohol (±)-**1a** (30 mg, 0.1 mmol, 1.0 eq.) in toluene (2 mL). The reaction mixture is stirred at 220 rpm at 30°C for 24 hours. After the reaction is complete, the crude product is filtered through a filtration plate, washing the enzyme several times with toluene. After concentrating the crude product under reduced pressure, the conversion was measured by NMR and the enantiomeric excess by HPLC. Results are presented in Table S1.

Table S1. Lipase-screening in the kinetic resolution of racemic (1-(quinolin-8-yl)naphthalen-2-yl)methanol **1a.**

Entry	Lipase	Time (h)	*ee* 1a (%)	*ee* 2a (%)	*c* (%)	*E*
**1**	CAL A	24	≤3	5	44	1
**2**	CAL B	24	59	97	38	120
**3**	PSL	72	≤3	≤3	≤3	n.d
**4**	CRL	72	≤3	6	≤3	n.d
**5**	PPL	24	≤3	≤3	≤3	n.d

n.d. not determined

2.2.1. Optimization of the reaction parameters in the kinetic resolution of alcohol (±)-1a.

To a suspension of CalB (30 mg) and alcohol (±)-**1a** (30 mg, 0.1 mmol) in the corresponding solvent (2.0 mL), vinyl acetate (29 µL, 3.0 eq.) was added. The reaction mixture was shaken at 220 rpm and 30ºC. Reaction progress was monitored by TLC, using a 1:1Hexane/EtOAc mixture as the eluent. After completion, CalB was filtered off, washing with Et_2O (10 mL). The filtrate was concentrated reduced pressure. Crude reaction was purified by flash chromatography using a *n*-hexane/EtOAc (1:1) mixture as the eluent. Table S2 shows the results achieved:

Table S2. Solvent effect in the KR of racemic alcohol **1a** catalyzed by CalB.

Entry	Solvent	*ee* 1a (%)	*ee* 2a (%)	*c* (%)	*E*
1	TBME	93	96	49	168
2	THF	22	82	22	13
3	Cyrene	≤3	≤3	≤3	1
4	CPME	94	95	50	139
5	2-MeTHF	≤3	≤3	≤3	1
6	1,4-dioxane	≤3	≤3	≤3	1
7	Et_2O[a]	67	96	41	99
8	Vinyl acetate[b]	34	97	26	92
9	Bu_2O[c]	≤3	≤3	≤3	1

2.2.2. CalB-recycling at the kinetic resolution of (±)-1a.

The racemic alcohol (30 mg, 0.1 mmol, 1.0 eq.) and CalB (30 mg) were mixed with CPME (2.0 mL). Vinyl acetate (29 µL, 0.3 mmol, 3.0 eq.) was then added (29 µL, 3.0 eq.) and the mixture was stirred at 220 rpm at the corresponding temperature (45 or 70ºC) using a heating block for 2.5 hours. After this reaction time, the reaction was cooled and filtrated under vacuum pressure employing a filtrate plate. The CalB was washed first with CPME followed by Et_2O. The enzyme was taken to dryness under reduced pressure for 1 hour before the next cycle. The conversion and the optical purity were measured by spectroscopy NMR and HPLC respectively. In Table S3, results of recycling are presented.

Table S3. CalB- recycling at the kinetic resolution of (±)-**1a** in CPME and vinyl acetate at 45º and 70ºC.

Entry	T (ºC)	Cycle	*ee* 1a (%)	*ee* 2a (%)	*c* (%)	*E*
1	45	1	99	95	51	>200
2	45	2	99	96	50	>200
3	45	3	99	96	50	>200
4	45	4	99	96	50	>200
5	45	5	85	95	47	105
6	45	6	40	95	30	58
7	70	1	73	97	45	200
8	70	2	48	97	43	144
9	70	3	24	97	33	106

10	70	4	23	97	20	100
11	70	5	73	97	19	91

2.3. General procedure for the kinetic resolution of heterobiaryl alcohols (±)-1b-i.

To the solution of the corresponding racemic alcohol **1b-i** (50 mM) in CPME (1.0 mL) in presence of the CalB (15 mg) at 70ºC and 220 rpm using a heating block, the vinyl acetate (3.0 eq.) was added. The reaction was stirred at 45ºC and 70ºC for 3 hours. After this time, the reaction was cooled to room temperature and filtrated. The crude of the reaction was evaporated under reduced pressure. Conversions were determined by NMR experiments and the enantiomeric excess were quantified employing HPLC. The final product was isolated by chromatography column using a *n*-Hexane/EtOAc (1:1) eluent.

Table S4. Kinetic Resolution of racemic heterobiaryl alcohols **1b-i** employing CalB and vinyl acetate at 45 and 70ºC**.**

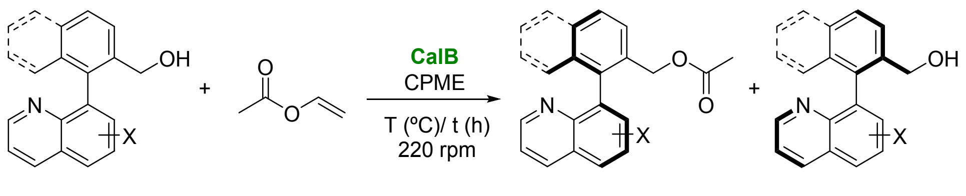

Entry	X	T (ºC)	t (h)	*ee* 1 (%)	*ee* 2 (%)	*c* (%)	*E*
1	5-CF_3 (**1b**)	45	15	97	97	50	>200
2	5-CF_3 (**1b**)	70	3	61	99	38	>200
3	5-Cl (**1c**)	45	5	69	99	41	>200
4	5-Cl (**1c**)	70	3	37	99	28	>200
5	6-F (**1d**)	45	5	51	97	34	109
6	6-F (**1d**)	70	3	35	97	32	102
7	6-Me (**1e**)	45	15	56	96	37	86
8	6-Me (**1e**)	70	3	20	97	17	80
9	H (**1f**)	45	15	99	96	50	>200
10	H (**1f**)	70	3	94	99	49	>200
11	5-Me (**1g**)	45	15	90	97	48	>200
12	5-Me (**1g**)	70	3	67	98	41	>200
13	5-F (**1h**)	45	15	98	95	50	179
14	5-F (**1h**)	70	3	75	97	44	149
15	5-Cl (**1i**)	45	15	96	99	49	>200
16	5-Cl (**1i**)	70	3	70	98	42	>200

(*R*)-(1-(quinolin-8-yl)naphthalen-2-yl)methyl acetate (2a). Following the general procedure of the kinetic resolution at 45ºC, (*R*)-**2a** was obtained as a yellow pale oil (7.3 mg, 45% yield). $[\alpha]_D^{20}$ = -17.8 (c 0.75, $CHCl_3$) for 99% *ee*. HPLC (IA column, 99:1 *n*-Hex/*i*-PrOH, 30 °C, 1.0 mL/min): t_R 21.9 min (*S*) and 29.2 min (*R*).

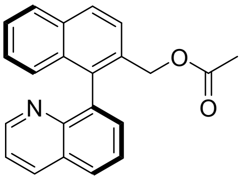

(*R*)-(1-(5-(trifluoromethyl)quinolin-8-yl)naphthalen-2-yl)methyl acetate (2b) . Following the general procedure of the kinetic resolution at 45ºC, (*R*)-**2b** was obtained as a yellow pale oil (7.1 mg, 39% yield). $[\alpha]_D^{20}$ = -13.4 (c 0.61, $CHCl_3$) for 97% *ee*. HPLC (IA column, 99:1 *n*-Hex/*i*-PrOH, 30 °C, 1.0 mL/min): t_R 11.1 min (*R*) and 13.5 min (*S*).

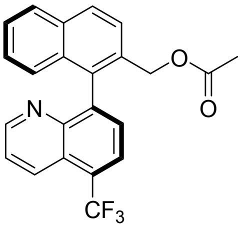

(*R*)-(1-(5-chloroquinolin-8-yl)naphthalen-2-yl)methyl acetate (2c). Following the general procedure of the kinetic resolution at 45ºC, (*R*)-**2c** was obtained as a yellow solid (6.1 mg, 37% yield). $[\alpha]_D^{20}$ = -28.0 (c 0.70, $CHCl_3$) for 99% *ee*. HPLC (IA column, 99:1 *n*-Hex/*i*-PrOH, 30 °C, 1.0 mL/min): t_R 15.2 min (*R*) and 18.1 min (*S*).

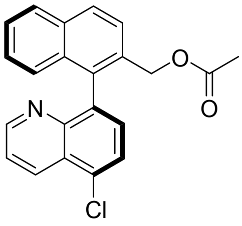

(*R*)-(1-(6-fluoroquinolin-8-yl)naphthalen-2-yl)methyl acetate (2d). Following the general procedure of the kinetic resolution at 45ºC, (*R*)-**2d** was obtained as a yellow pale oil (5.2 mg, 30% yield). $[\alpha]_D^{20}$ = -14.6 (c 0.58, $CHCl_3$) for 99% *ee*. HPLC (IA column, 99:1 *n*-Hex/*i*-PrOH, 30 °C, 1.0 mL/min): t_R 14.2 min (*R*) and 16.1 min (*S*).

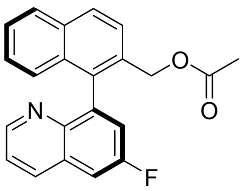

(*R*)-(1-(6-methylquinolin-8-yl)naphthalen-2-yl)methyl acetate (2e). Following the general procedure of the kinetic resolution at 45ºC, (*R*)-**2e** was obtained as a yellow pale oil (5.6 mg, 33% yield). $[\alpha]_D^{20}$ = -21.6 (c 0.47, $CHCl_3$) for 96% *ee*. HPLC (IA column, 99:1 *n*-Hex/*i*-PrOH, 30 °C, 1.0 mL/min): t_R 21.0 min (*R*) and 24.9 min (*S*).

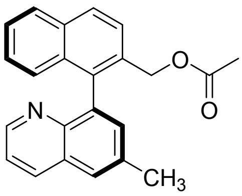

(*R*)-3-methyl-2-(quinolin-8-yl)benzyl acetate (2f). Following the general procedure of the kinetic resolution at 45ºC, (*R*)-**2f** was obtained as a yellow pale oil (5.6 mg, 37% yield). $[\alpha]_D^{20}$ = -12.3 (c 0.45, $CHCl_3$) for 96% *ee*. HPLC (IC column, 95:5 *n*-Hex/*i*-PrOH, 30 °C, 1.0 mL/min): t_R 17.9 min (*S*) and 22.9 min (*R*).

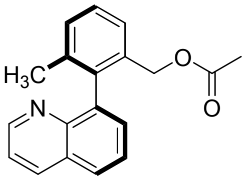

(*R*)-3-methyl-2-(quinolin-8-yl)benzyl acetate (2g). Following the general procedure of the kinetic resolution at 70ºC, (*R*)-**2g** was obtained as a yellow pale oil (6.1 mg, 39% yield). $[\alpha]_D^{20}$ = -15.7 (c 0.5, $CHCl_3$) for 98% *ee*. HPLC (IC column, 97:3 *n*-Hex/*i*-PrOH, 30 °C, 1.0 mL/min): t_R 27.0 min (*S*) and 29.7 min (*R*).

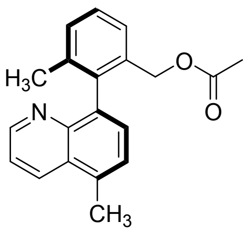

(*R*)-2-(5-fluoroquinolin-8-yl)-3-methylbenzyl acetate (2h). Following the general procedure of the kinetic resolution at 70ºC, (*R*)-**2h** was obtained as a yellow pale oil (6.0 mg, 39% yield). $[\alpha]_D^{20}$ = -18.1 (c 0.58, $CHCl_3$) for 97% *ee*. HPLC (IC column, 99:1 *n*-Hex/*i*-PrOH, 30 °C, 1.0 mL/min): t_R 32.5 min (*S*) and 37.8 min (*R*).

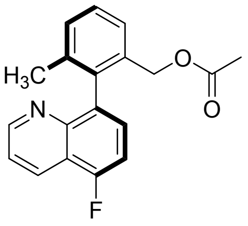

(*R*)-2-(5-chloroquinolin-8-yl)-3-methylbenzyl acetate (2i). Following the general procedure of the kinetic resolution at 70ºC, (*R*)-**2i** was obtained as a yellow pale oil (6.0 mg, 39% yield). $[\alpha]_D^{20}$ = -24.6 (c 1.07, $CHCl_3$) for 98% *ee*. HPLC (IC column, 99:1 *n*-Hex/*i*-PrOH, 30 °C, 1.0 mL/min): t_R 30.4 min (*S*) and 32.8 min (*R*).

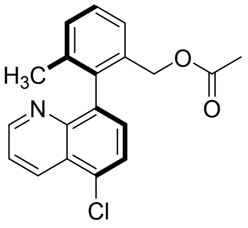

2.4. General procedure for the Dynamic Kinetic Resolution of racemic heterobiaryl alcohols 1a-i in presence of CalB and metal catalysts (I-IX).

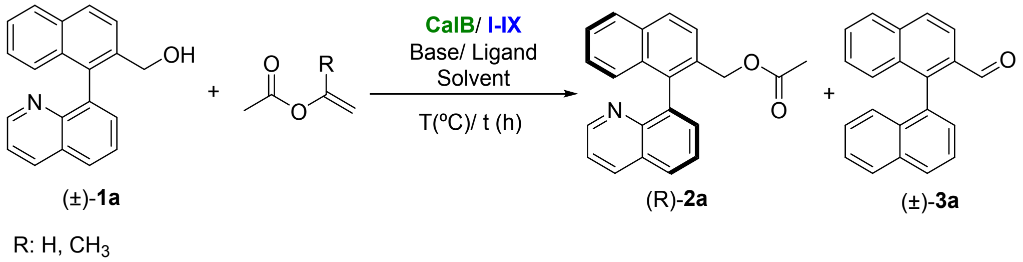

In a flamed Schlenk-tube and under inert atmosphere, to a mixture of the corresponding metal catalyst (2-20 mol%), the selected ligand (if necessary, 5-20 mol%), the corresponding base (if necessary) in CPME (2 mL), racemic alcohol **1a** (30 mg, 0.1 mmol) was added followed by vinyl or isopropenyl acetate (29 µL, 3.0 eq.). The reaction mixture was stirred at the selected temperature using a heating block and 220 rpm, controlling the progress by TLC. Once the reaction is finished, the crude was filtered off, concentrated under reduced pressure and purified by column chromatography .

2.4.1. DKRs of alcohols (±)-1a-i employing CalB and 1-hydroxytetraphenyl-cyclopentadienyl(tetraphenyl-2,4-cyclopentadien-1-one)-µ-hydrotetracarbonyldiruthenium(II) (Shvo's catalyst, I)

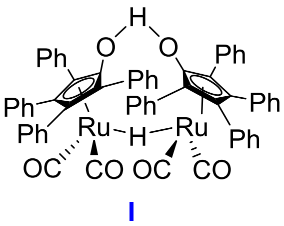

The followed procedure was adapted from Bäckvall and coworkers.[2] In a flamed Schlenk-tube and under argon atmosphere, the mixture of **I** (2.3 mg, 2 mol%) and CalB (30 mg) in the corresponding solvent (2.0 mL) was heated at 70ºC for 15 minutes. Then the racemic alcohol **1a-i** (0.1 mmol) was added followed by the acyl donor [vinyl acetate (29 µL, 3.0 eq.) or isopropenyl acetate (35 µL, 3.0 eq.)]. The reaction mixture was stirred at 70ºC using a heating block and 220 rpm till the reaction was complete, following the reaction progress by ^{1}H-NMR. The reaction mixture was filtered and concentrated under reduced pressure. Conversions and enantiomeric excesses were determined by NMR and HPLC analyses respectively. For the optimized conditions employing CPME and isopropenyl acetate, product purification was carried out by column chromatography, employing *n*-hexane:EtOAc (1:1) as eluent to obtain the corresponding chiral esters (*R*)-**2a-i** (isolated yields are given in Table S5).

Table S5. DKR of heterobiaryl alcohols (±)-**1a-i** employing CalB and Shvo's catalyst **(I).**

**Entry**	**Alcohol**	**t (h)**	**Solvent**	**Acetate**	**3a (%)**	**2a (%)**	**Yield 2a (%) / (mg)**	***ee* 2a (%)**
1	**1a**	48	CPME	Vinyl	6	94	n.d.	80
2	**1a**	48	Toluene	Vinyl	16	84	n.d.	77

3	**1a**	48	1,4-dioxane	Vinyl	27	73	n.d.	75
4	**1a**	24	CPME	Isopropenyl	3	97	90 / 29.4 mg	96
5	**1b**	24	CPME	Isopropenyl	6	94	89 / 35.1 mg	98
6	**1c**	24	CPME	Isopropenyl	13	87	81 / 29.2 mg	95
7	**1d**	24	CPME	Isopropenyl	3	75	67 / 23.1 mg	94
8	**1e**	24	CPME	Isopropenyl	21	79	72 / 24.5 mg	90
9	**1f**	24	CPME	Isopropenyl	5	95	90 / 26.2 mg	96
10	**1g**	24	CPME	Isopropenyl	13	87	80 / 24.4 mg	98
11	**1h**	24	CPME	Isopropenyl	3	97	93 / 28.7 mg	98
12	**1i**	24	CPME	Isopropenyl	3	97	93 / 30.2 mg	97

n.d. not determined.

2.4.2. DKR or racemic alcohol (±)-1a in presence of lipase CalB and chlorodicarbonyl(1,2,3,4,5-pentaphenylcyclopentadienyl)ruthenium(II) (Bäckvall´s catalyst, II)

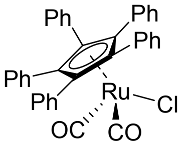

II

The present DKR process was adapted to a 50 mM scale from the descripted procedure in literature.[3] The mixture of **II** (5 mol%, 3.4 mg), CalB (30 mg), Na_2CO_3 (10.6 mg, 1.0 eq.) and ^{t}BuOK solution (2.5 M in *n*-hexane, 5 µL, 5.0 mol%) in CPME (2.0 mL) was stirred, under argon atmosphere, at room temperature for 6 min. Then alcohol **1a** (30 mg, 0.1 mmol) was added, and after 4 min, vinyl acetate (29 µL, 3.0 eq.) was added. The reaction mixture was stirred at the selected temperature using a heating block and 220 rpm. The reaction progress was controlled by TLC and NMR experiments of aliquots at different reaction times. Once the reaction was finished, the crude was filtrated and evaporated under reduced pressure. Conversions were determined by NMR methods and the enantiomeric excess were evaluated by HPLC. Purification was carried out in a chromatography column employing a *n*-Hexane/EtOAc (2:1) mixture. Results are presented in Table S6.

Table S6. DKR of heterobiaryl alcohol **1a** employing Bäckvall’s catalyst **(II)** and CalB.

Entry	II [mol%]	Time (h)	Acetate	T (ºC)	2a (%)	3a (%)	*ee* 2a (%)
**1**	5	48	Vinyl	30	35	4	96
**2**[a]	7.5	96	Isopropenyl	70	83	6	69
**3**	7.5	96	Isopropenyl	70	64	15	89
**4**	10	72	Vinyl	70	45	17	87

**5**	10	48	Vinyl	45	56	8	92
**6**[b]	10	72	Vinyl	70	27	11	89
**7**[c]	10	72	Vinyl	70	76	0	84
**8**[d]	10	72	Vinyl	70	57	6	83
**9**[c]	10	96	Isopropenyl	70	50	50	85

[a] Toluene employed as a solvent. [b] CPME/Toluene (4:1) was employed as solvent. [c] CPME/Toluene (1:1) was used as solvent. [d] CPME/Toluene (1:4) was used.

2.4.3. DKR of (±)-1a employing CalB and chloro(indenyl)bis(triphenylphosphine)ruthenium (III)

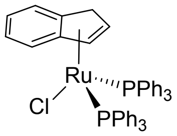

III

The followed procedure was adapted from Park and coworkers[4] to a 50 mM scale. A solution of **III** (4 mg, 5 mol%) and Et_3N (44 μL, 3.0 eq.) in CPME (2.0 mL) was stirred at 60ºC for 15 min. Then the CalB (30 mg) and alcohol **1a** (30 mg, 0.1 mmol) were added. Finally, vinyl acetate (29 μL, 3.0 eq.) was added. The reaction mixture was stirred at 60ºC using a heating block and 220 rpm, controlling the progress of the reaction by TLC and NMR experiments. After 24 hours, the crude of reaction was filtered and concentred. A 70% of **2a** was measured by ^{1}H-NMR and a 76% enantiomeric excess of (*R*)-**2a** product was observed by HPLC.

2.4.4. DKR of (±)-1a in presence of CalB and chloro(pentamethylcyclopentadienyl)(cyclooctadiene)ruthenium(II) (IV)

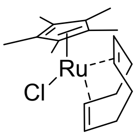

IV

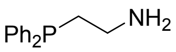

L1

The method followed was based on the approach developed by Ito *et al.*[5] Over a flamed Schlenck-tube and under Ar atmosphere, CalB (30 mg), **IV** (8 mg, 20 mol%) and **L_1** (5 μL, 20 mol%) were dissolved in CPME (2.0 mL). Next, ^{t}BuOK (20 μL, 20 mol%) was added, stirring the solution for 15 min at 30ºC or 60ºC using a heating block. Finally, the racemic alcohol **1a** (30 mg, 0.1 mmol) and vinyl acetate (29 μL, 3.0 eq.) were subsequently added to the mixture. The reaction mixture was stirred at the selected temperature and 220 rpm, tracking the reaction development through TLC and NMR analyses. The reaction crude was filtered and concentrated after 48 hours. No reaction was observed at 60ºC and only 26% conversion was achieved by ^{1}H-NMR analysis with 80% *ee* of (*R*)-**2a** at 30ºC.

2.4.5. DKR of (±)-1a employing CalB and pentamethylcyclopentadienyliridium(III) chloride dimer (V).

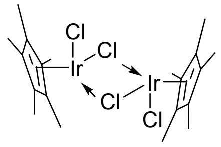

V

The procedure applied was derived from the methodology outlined by Fuyita and coworkers.[6] In a flame-dried Schlenk tube under an argon atmosphere, racemic alcohol **1a** (30 mg, 0.1 mmol), **cat. V** (4 mg, 5 mol%), K_2CO_3 (0.7 mg, 5 mol%), CalB (30 mg) and CPME (2.0 mL) were added. Next, vinyl acetate (29 µL, 3.0 eq.) was introduced. The reaction mixture was stirred at 100ºC using a heating block and 220 rpm monitoring the progress by TLC and NMR analyses. After 72 hours, the reaction crude was filtered and concentrated under reduced pressure, obtaining a 62% conversion and 68% *ee* of (*R*)-**2a**.

2.4.6. DKR of (±)-1a in presence of CalB and benzeneruthenium(II) chloride dimer (VI) or dichloro(1,5-cyclooctadiene)ruthenium(II) polymer (VII).

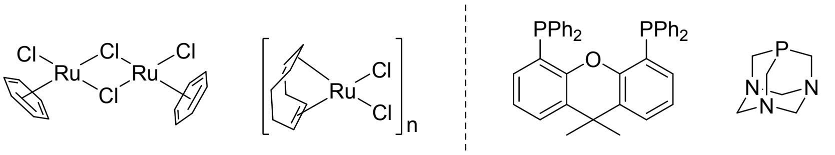

VI VII L2 (XANTPHOS) L3 (PTA)

This protocol was adapted from the study conducted by Taddei and coworkers.[7] Under an argon atmosphere in a flame-heated Schle V. R. Jumde, L. Gonsalvi, A. Guerriero, M. Peruzzini, M. Taddei, Eur. J. Org. Chem. 2015, 1829–1833.nk tube, **VI** (2.60 mg, 5 mol%) or **VII** (2.0 mg, 5 mol%), $\mathbf{L_2}$ (6.0 mg, 10 mol%) or $\mathbf{L_3}$ (2.0 mg, 10 mol%) were dissolved in CPME (2.0 mL). Then ^{t}BuOK (5.0 µL, 5 mol%) was added followed by CalB (30 mg). Finally, racemic alcohol **1a** (30 mg, 0.1 mmol) was introduced followed by vinyl acetate (29 µL, 3.0 eq.). The reaction mixture was stirred at the selected temperature (30ºC or 60ºC) using a heating block and 220 rpm. The progress of the reaction was determined by TLC. Once finished, the crude was filtrated and concentrated. Results are presented in Table S7, showing that no effective DKR occurred, a KR was observed.

Table S7. DKR of racemic heterobiaryl alcohol **1a** employing CalB and metal catalysts **VI** or **VII.**

Entry	Cat.	Ligand	Time (h)	T (ºC)	2a (%)	*ee* (*R*)-2a (%)
1	**VI**	$\mathbf{L_2}$	48	30	50	97
2	**VI**	$\mathbf{L_3}$	48	30	50	97
3	**VI**	$\mathbf{L_2}$	96	60	53	82
4	**VI**	$\mathbf{L_3}$	96	60	52	85
5	**VII**	$\mathbf{L_2}$	48	30	50	97

6	**VII**	**L_3**	48	30	50	96
7	**VII**	**L_2**	96	60	50	91
8	**VII**	**L_3**	96	60	56	84

2.4.7. DKR of (±)-1a in presence of CalB and trimethylaluminum (VIII)

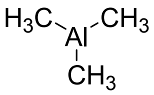

VIII

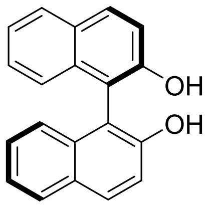

L_4 [(*R*)-BINOL]

The adopted procedure was modified from that described by Berkessel *et al.*[8] Under argon atmosphere in a flamed schlenck-tube, **L_4** (3.0 mg, 10 mol%) and **VIII** (5.3 µL, 10 mol%) were dissolved in CPME (2 mL). The mixture was stirred at the 30ºC for 15 min in a rotatory shaker at 220 rpm. Then the racemic alcohol **1a** (30 mg, 0.1 mmol) was added and stirred for 5 minutes. Finally, CalB (30 mg) and vinyl acetate (29 µL, 3.0 eq.) were introduced. The reaction mixture was stirred at 30ºC and 220 rpm, controlling the progress by TLC and NMR analyses. The crude of the reaction was filtrated and concentrated. Results are presented in Table S8.

Table S8. DKR results of heterobiaryl alcohol **1a** employing **VIII** and CalB**.**

Entry	Time (h)	T (ºC)	2a (%)	*ee* (*R*)-2a (%)
1	24	30	57	98
2	24	60	55	81
3	48	60	61	70
4	72	60	64	60

2.4.8. DKR of (±)-1a in presence of CalB and vanadium(IV) oxide sulfate hydrate (IX)

$VOSO_4 \cdot xH_2O$

IX

The followed procedure was adapted from Milagre and coworkers.[9] To the mixture of CalB (30 mg), **IX** (50 mg, 3.0 eq.) and the racemic alcohol **1a** (30 mg, 0.1 mmol), vinyl acetate (29 µL, 3.0 eq.) was added. The mixture was stirred at 60ºC using a heating block for 96 hours, not observing any reaction after this time.

2.5. DKR of (±)-1a employing CalB and Shvo's catalyst at high scale

In a flamed Schlenk-tube and under argon atmosphere, a mixture of **I** (45.6 mg, 2 mol%) and CalB (500 mg) in CPME (40 mL) was heated at 70ºC for 15 minutes. Then the racemic alcohol **1a** (600 mg, 2.10 mmol) was added followed by isopropenyl acetate (700 µL, 3.15 mmol). The reaction mixture was stirred at 70ºC using a heating block and 220 rpm for 24 hours until completion (followed by TLC using n-hexane:EtOAc 1:1 as eluent). The enzyme was filtered

off, washing with diethyl ether (3 x 15 mL) and concentrated under reduced pressure. The crude residue was purified by flash chromatography, employing *n*-hexane:EtOAc (1:1) as eluent to obtain the corresponding chiral esters (*R*)-**2a** (604.0 mg, 88% yield) as a yellow pale oil with 97% enantiomeric excess.

3. References

(1) Rodríguez-Franco, C.; Roldán-Molina, E.; Aguirre-Medina, A.; Fernández, R.; Hornillos, V.; Lassaletta, J. M. Catalytic Atroposelective Synthesis of C−N Axially Chiral Aminophosphines *via* Dynamic Kinetic Resolution. *Angew. Chem. Int. Ed.* **2024**, *63*, e202409524.

(2) Persson, B. A.; Larsson, A. L. E.; Ray, M. L.; Bäckvall, J.-E. Ruthenium- and Enzyme-Catalyzed Dynamic Kinetic Resolution of Secondary Alcohols. *J. Am. Chem. Soc.* **1999**, *121*, 1645-1650.

(3) Martín-Matute, B.; Edin, M.; Bogár, K.; Bëtul Kaynak, F.; Bäckvall, J.-E. Combined Ruthenium(II) and Lipase Catalysis for Efficient Dynamic Kinetic Resolution of Secondary Alcohols. Insight into the Racemization Mechanism. *J. Am. Chem. Soc.* **2005**, *127*, 8817-8825.

(4) Koh, J. H.; Jung, H. M.; Kim, M.; Park, J. Enzymatic Resolution of Secondary Alcohols Coupled with Ruthenium-Catalyzed Racemization without Hydrogen Mediator. *Tetrahedron Lett.* **1999**, *40*, 6281-6284.

(5) Ito, M.; Osaku, A.; Kitahara, S.; Hirakawa, M.; Ikariya, T. Rapid Racemization of Chiral non-Racemic *sec*-Alcohols Catalyzed by (η^5-$C_5(CH_3)_5$)Ru Complexes Bearing Tertiary Phosphine–Primary Amine Chelate Ligands. *Tetrahedron Lett.* **2003**, *44*, 7521-7523.

(6) Fujita, K.; Li, Z.; Ozeki, N.; Yamaguchi, R. N-Alkylation of Amines with Alcohols Catalyzed by a Cp*Ir Complex. *Tetrahedron Lett.* **2003**, *44*, 2687-2690.

(7) Jumde, V. R.; Gonsalvi, L.; Guerriero, A.; Peruzzini, M.; Taddei, M. A Ruthenium-Based Catalytic System for a Mild Borrowing-Hydrogen Process. *Eur. J. Org. Chem.* **2015**, 1829–1833.

(8) Berkessel, A.; Sebastian-Ibarz, M. L.; Müller, T. N. Dynamic Kinetic Resolution of Alcohols by Enantioselective Silylation Enabled by Two Orthogonal Transition-Metal Catalysts. *Angew. Chem. Int. Ed.* **2006**, *45*, 6567-6570.

(9) De Almeida, L. A.; Marcondes, T. H.; Milagre, C. D. F.; Milagre, H. M. S. Lipase-Oxovanadium Heterogeneous Catalysis System: a Robust Protocol for the Dynamic Kinetic Resolution of *sec*-Alcohols. *ChemCatChem* **2020**, *12*, 2849-2858.

4. NMR and HPLC spectra of compounds 2a-i

^{1}H NMR (500 MHz, $CDCl_3$) of (±)-**2a**:

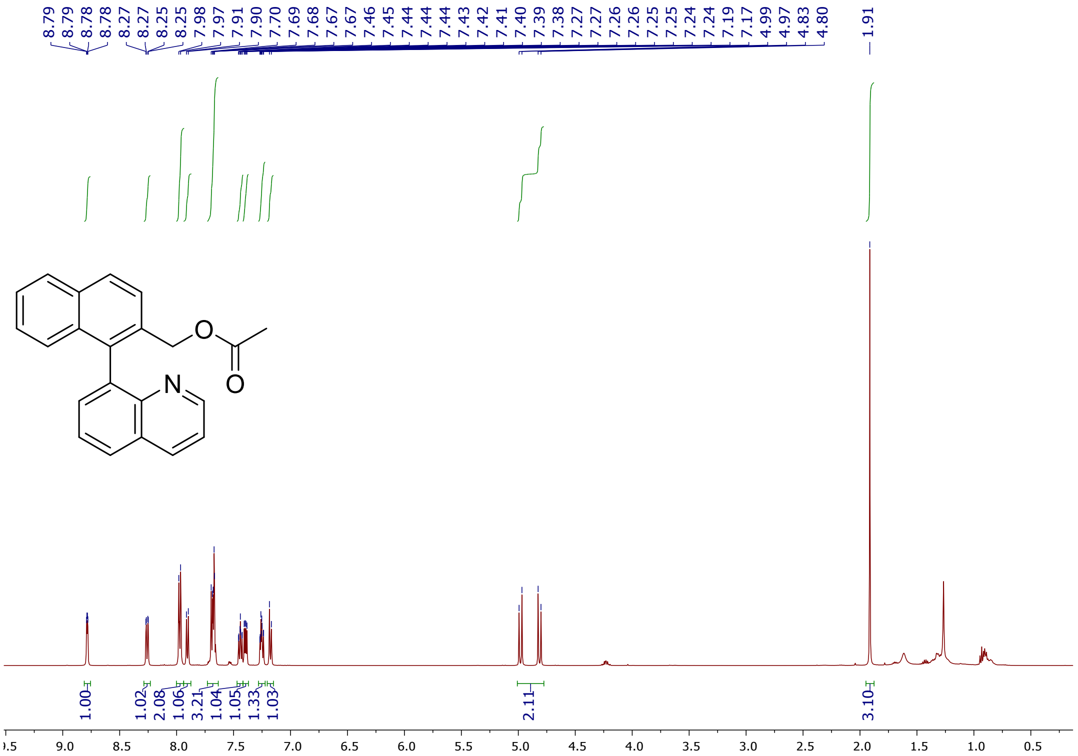

^{13}C-NMR{^{1}H} ($CDCl_3$, 125 MHz) of **2a.**

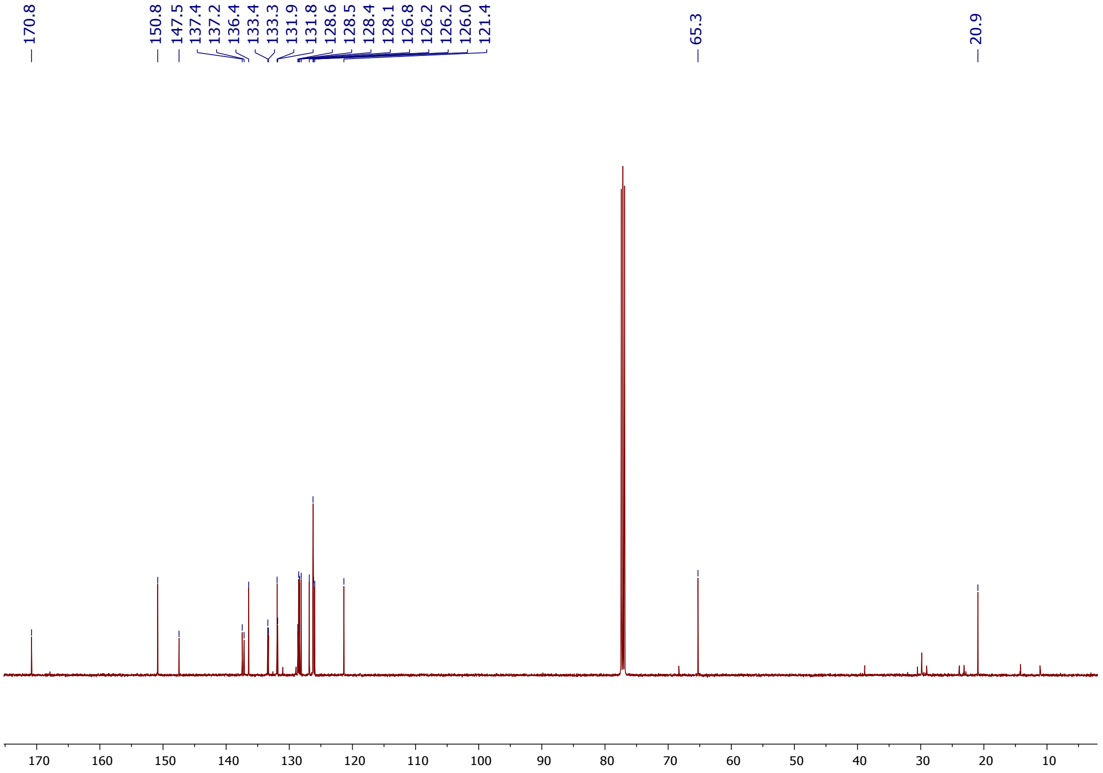

Racemic sample of **2a:** IC column, *n*-Hex/*i*-PrOH 95:5, T = 30 ºC, F = 1.0 mL/min.

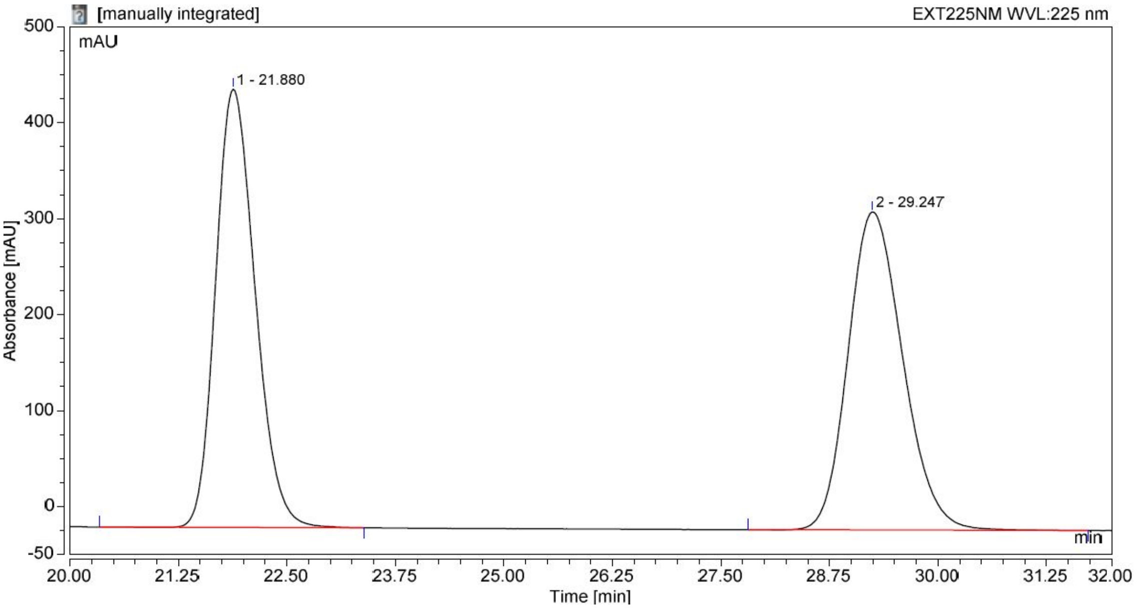

Integration Results			
No.	Retention Time min	Area mAU*min	Relative Area %
1	21.880	240.774	49.87
2	29.247	242.012	50.13

Enantioenriched sample of (*R*)-**2a:**

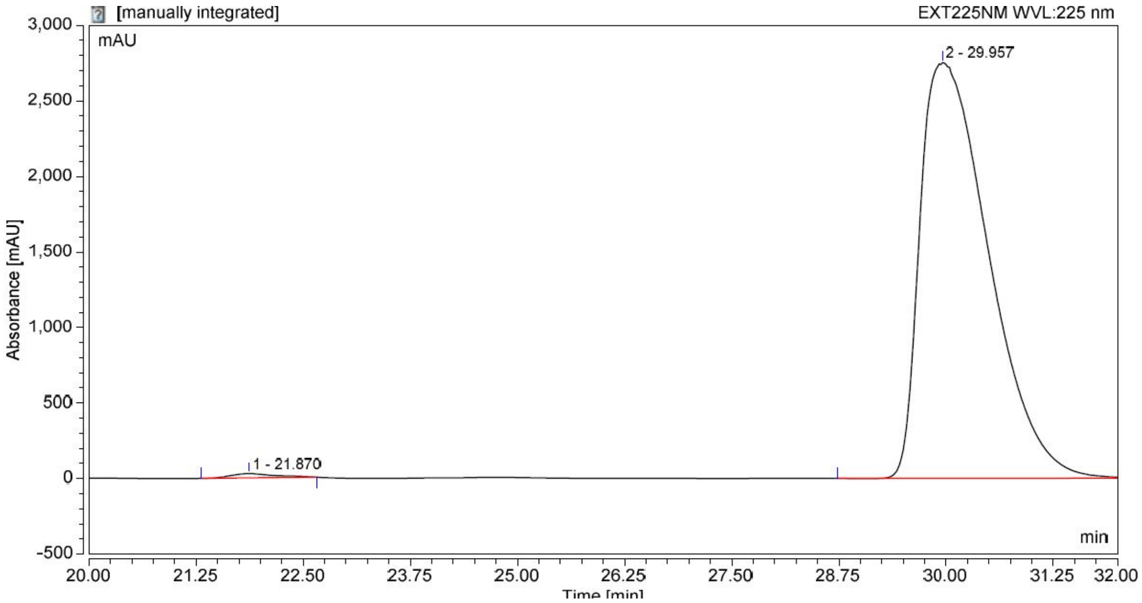

Integration Results			
No.	Retention Time min	Area mAU*min	Relative Area %
1	21.870	17.484	0.68
2	29.957	2555.730	99.32

^{1}H NMR (500 MHz, $CDCl_3$) of (±)-**2b**:

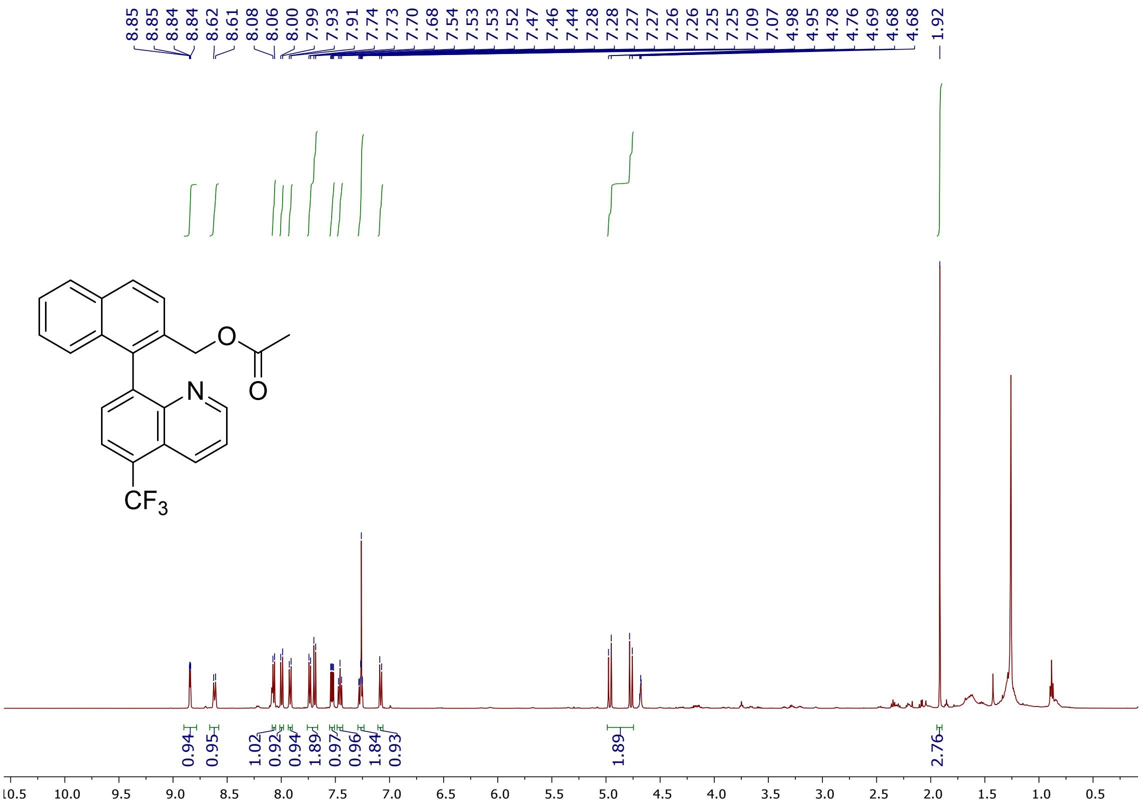

^{13}C NMR{^{1}H} (125 MHz, $CDCl_3$) of (±)-**2b**:

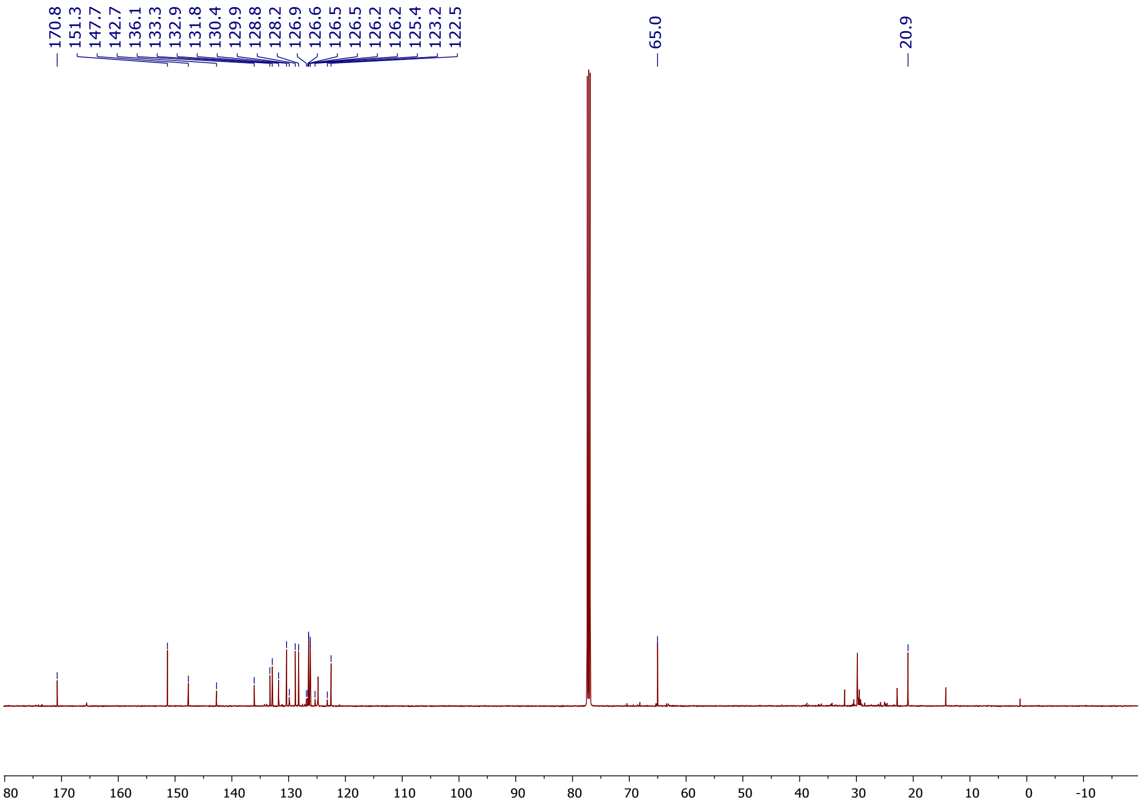

^{19}F NMR (471 MHz, $CDCl_3$) of (±)-**2b**:

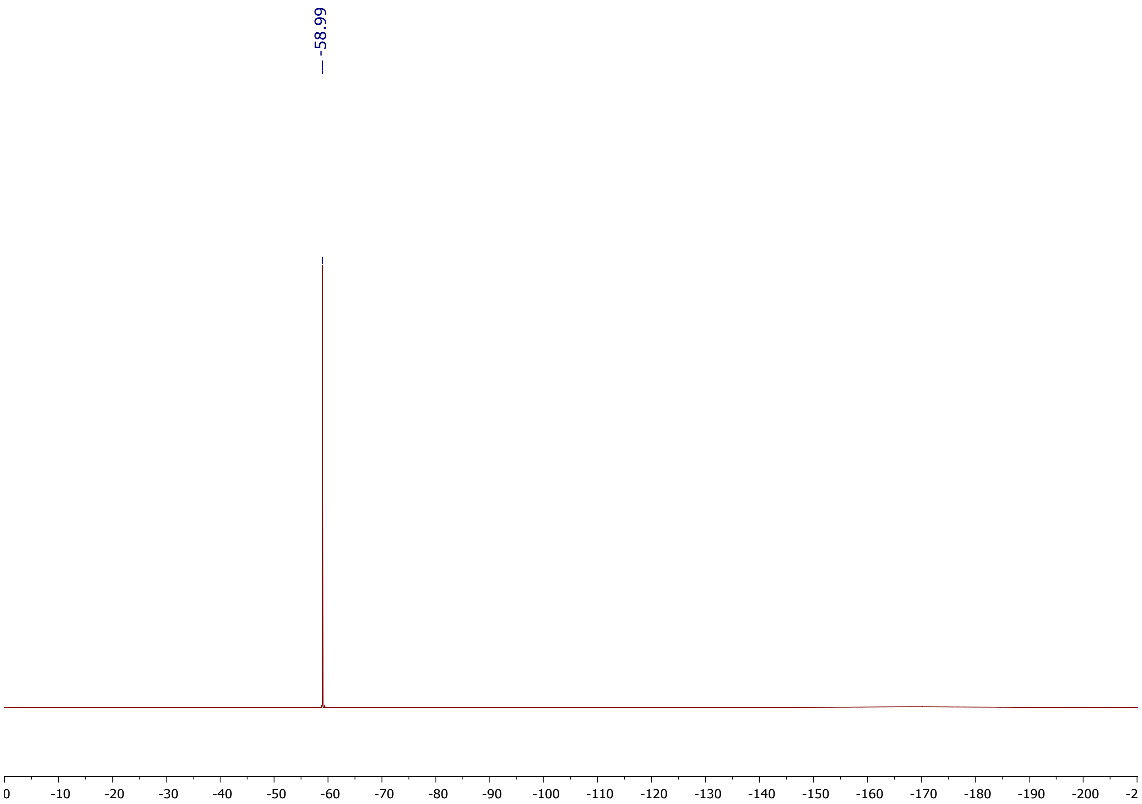

Racemic sample of **2b**: IA column, *n*-Hex/*i*-PrOH 99:1, T = 30 °C, F = 1 mL/min.

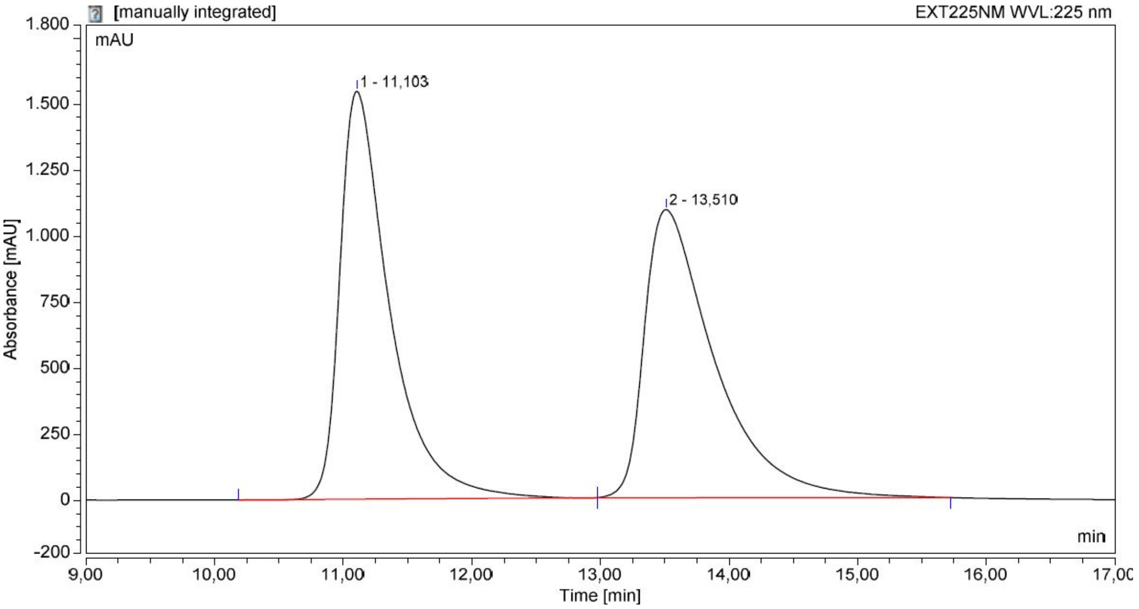

Integration Results			
No.	Retention Time min	Area mAU*min	Relative Area %
1	11,103	693,553	50,15
2	13,510	689,435	49,85

Enantioenriched sample of (*R*)-**2b**:

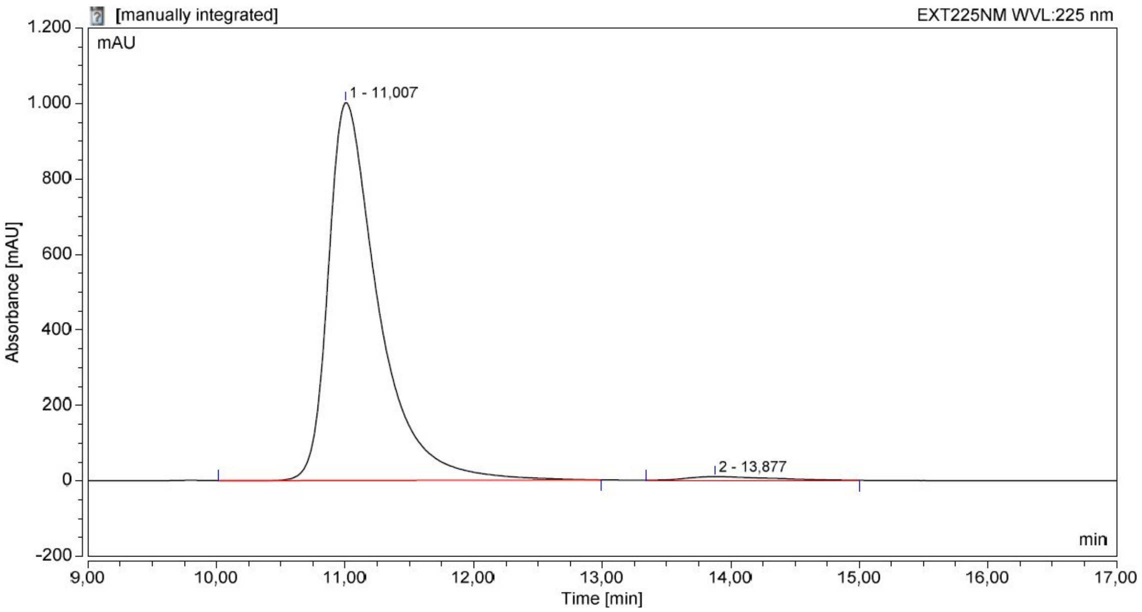

Integration Results			
No.	Retention Time<br>min	Area<br>mAU*min	Relative Area<br>%
1	11,007	451,641	98,45
2	13,877	7,125	1,55

^{1}H NMR (500 MHz, $CDCl_3$) of (±)**2c**:

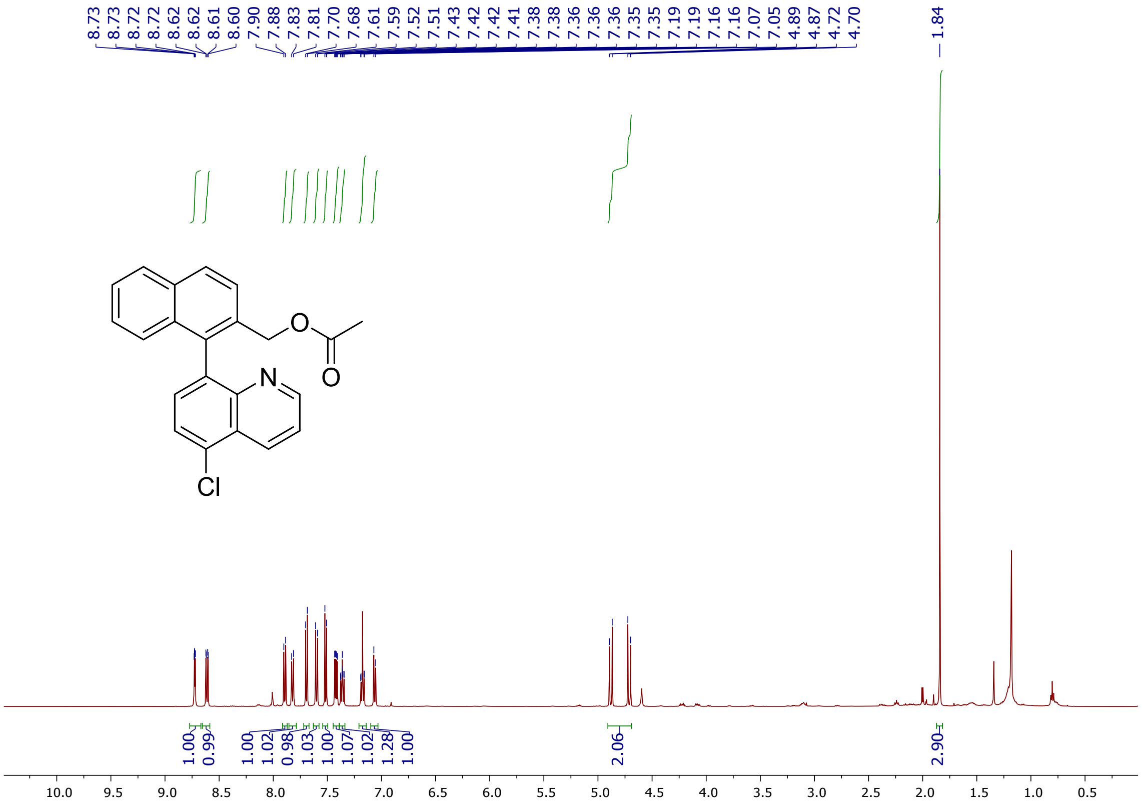

^{13}C NMR{^{1}H} (125 MHz, $CDCl_3$) of (±)-**2c**:

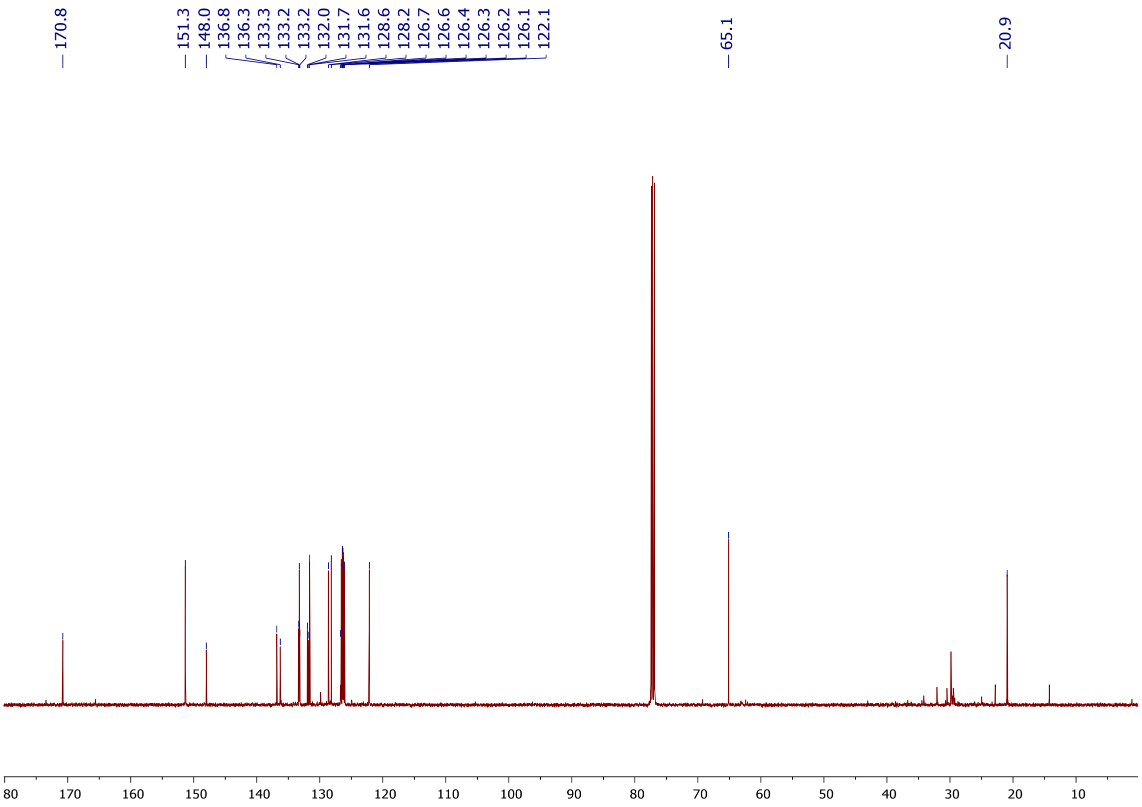

Racemic sample of **2c**: IA column, *n*-Hex/*i*-PrOH 99:1, T = 30 °C, F = 1 mL/min.

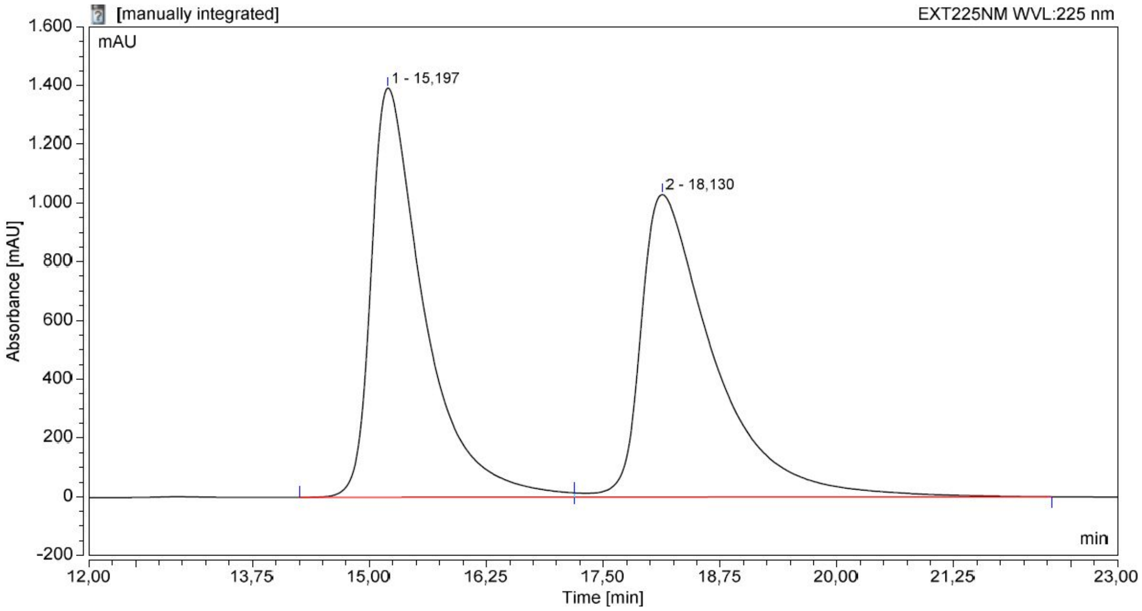

Integration Results			
No.	Retention Time min	Area mAU*min	Relative Area %
1	15,197	915,064	49,51
2	18,130	933,067	50,49

Enantioenriched sample of (*R*)-**2c**:

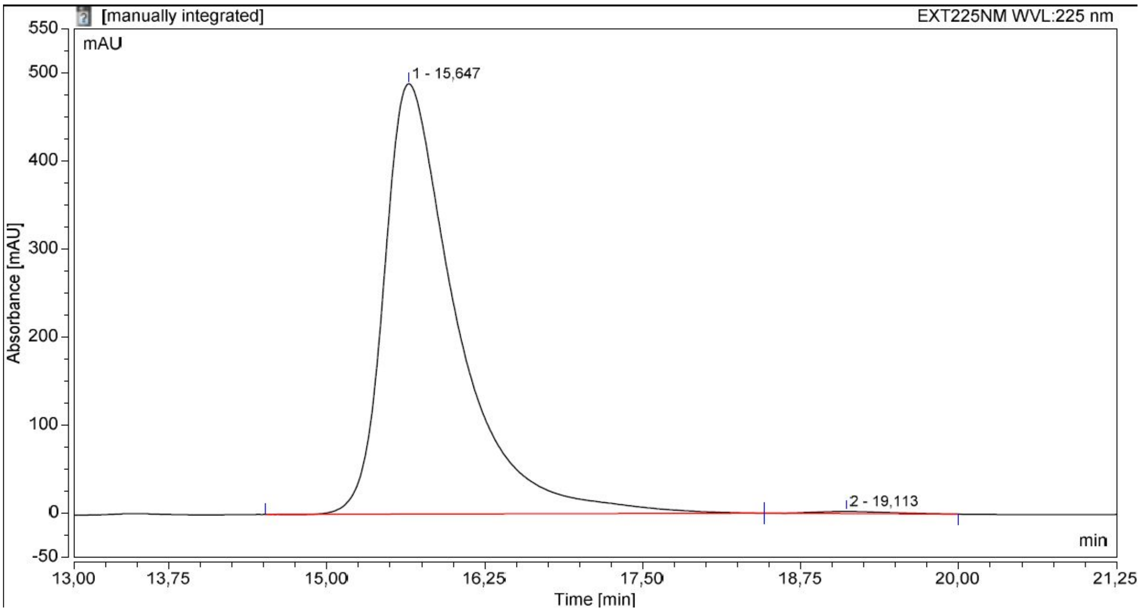

Integration Results			
No.	Retention Time min	Area mAU*min	Relative Area %
1	15,647	325,567	99,49
2	19,113	1,660	0,51

^{1}H NMR (500 MHz, $CDCl_3$) of (±)**2d**:

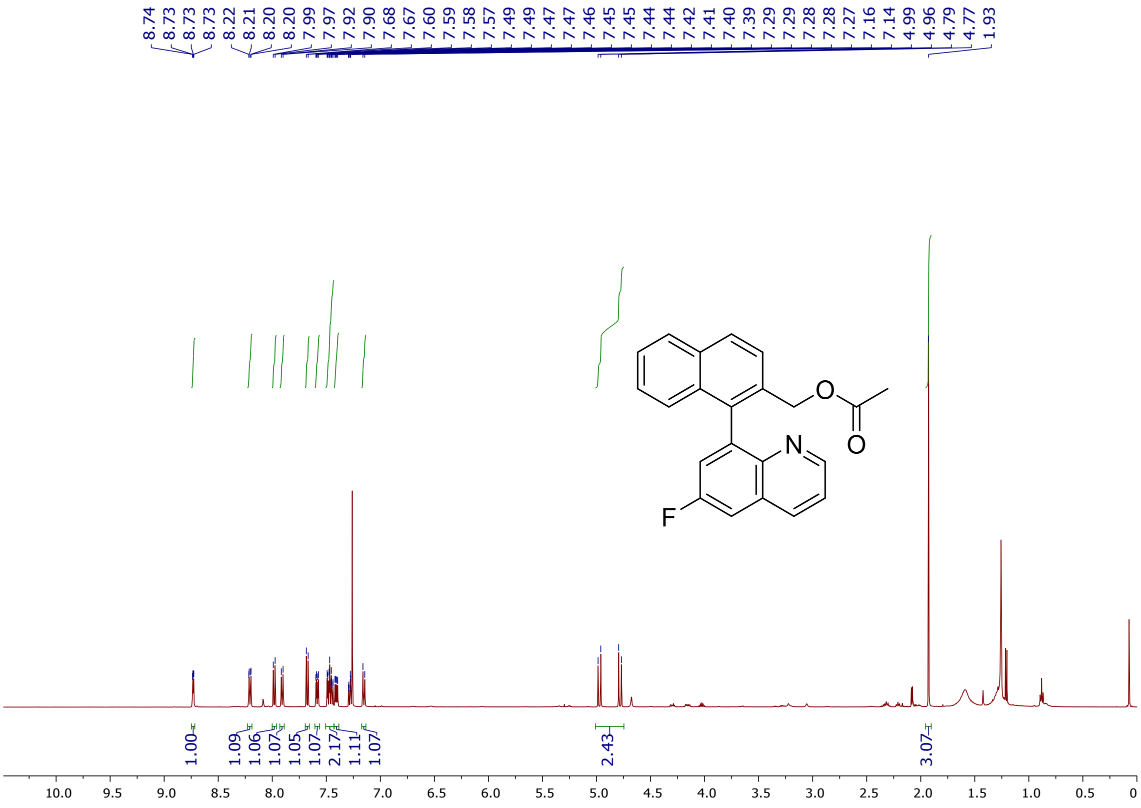

^{13}C NMR{^{1}H} (125 MHz, $CDCl_3$) of (±)-**2d**:

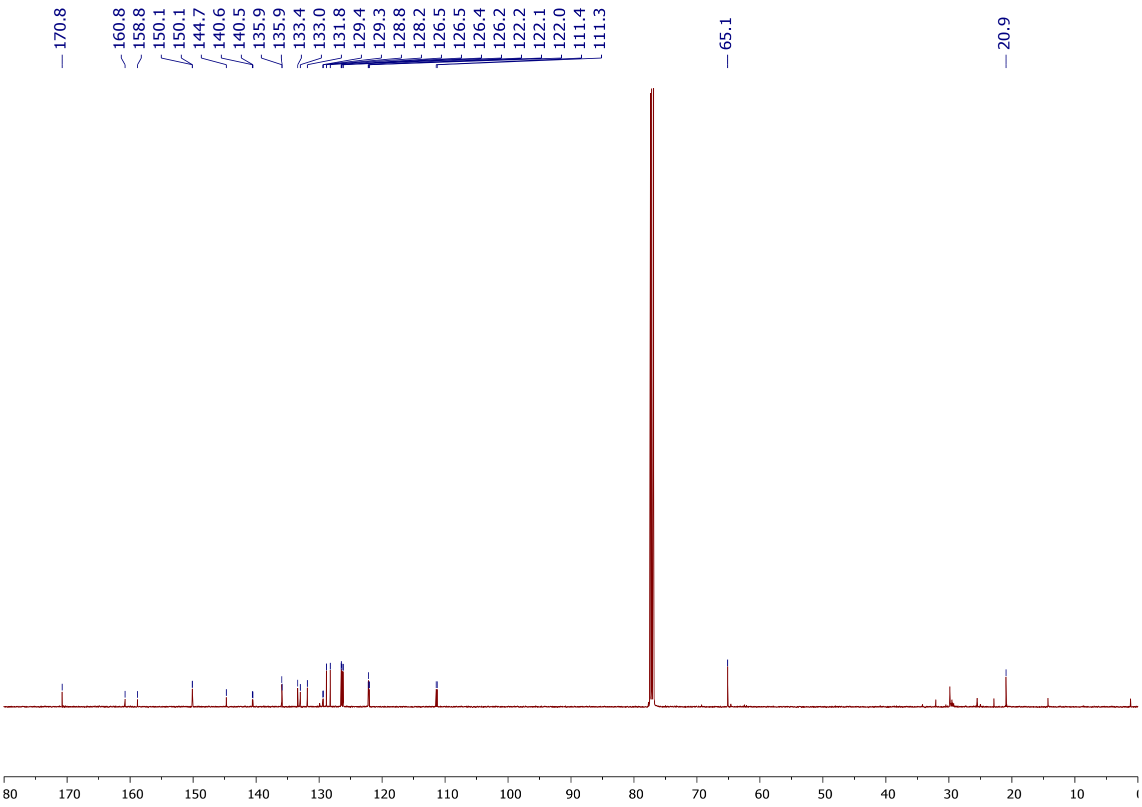

^{19}F NMR (471 MHz, $CDCl_3$) of (±)-**2d**:

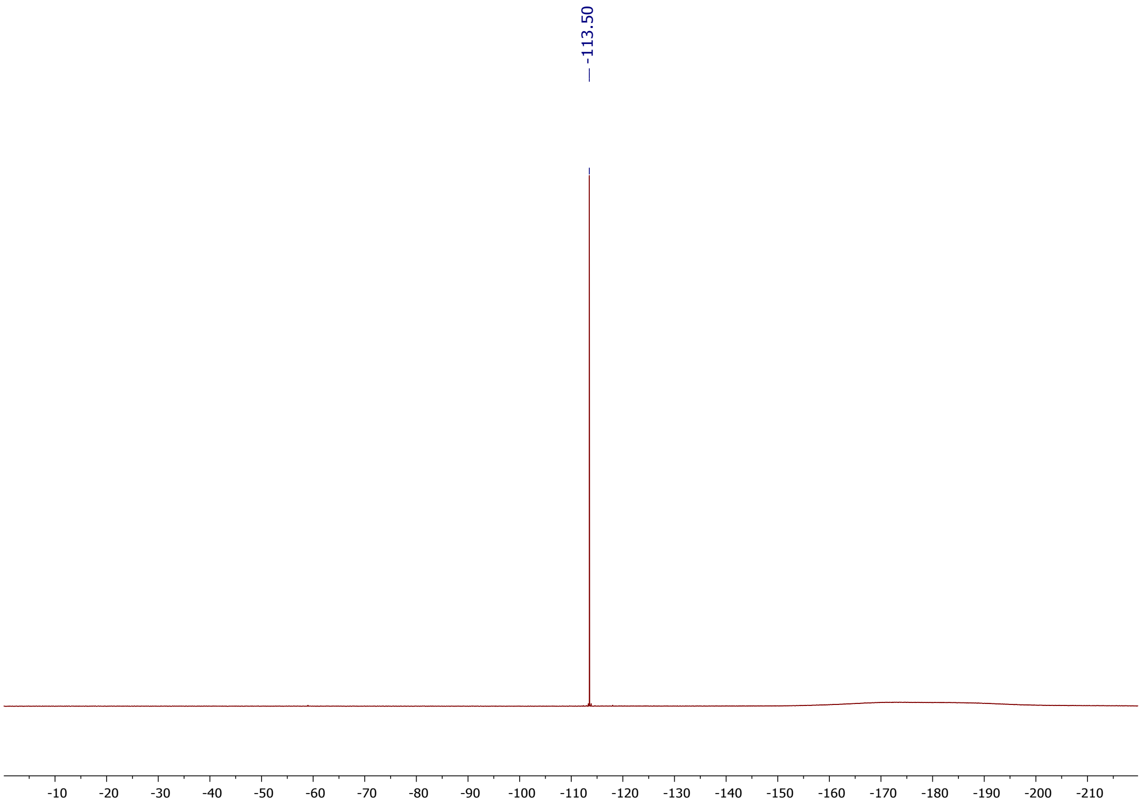

Racemic sample of **2d**: IA column, *n*-Hex/*i*-PrOH 99:1, T = 30 °C, F = 1 mL/min.

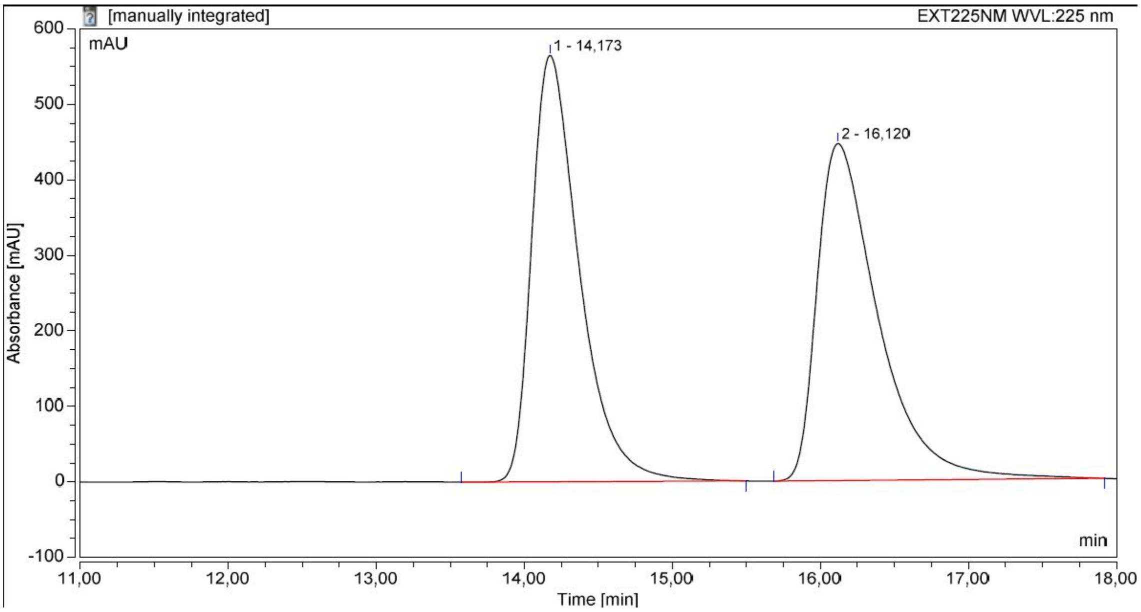

Integration Results			
No.	Retention Time<br>min	Area<br>mAU*min	Relative Area<br>%
1	14,173	210,865	49,66
2	16,120	213,725	50,34

Enantioenriched sample of (*R*)-**2d**:

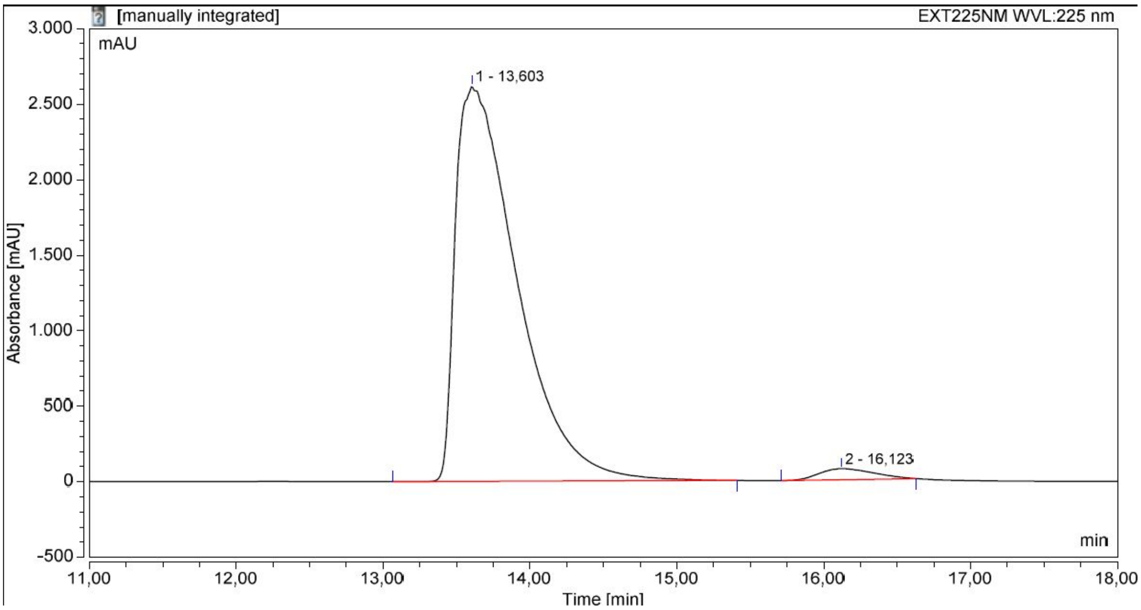

Integration Results			
No.	Retention Time<br>min	Area<br>mAU*min	Relative Area<br>%
1	13,603	1268,835	97,62
2	16,123	30,976	2,38

^{1}H NMR (500 MHz, $CDCl_3$) of (±)**2e**:

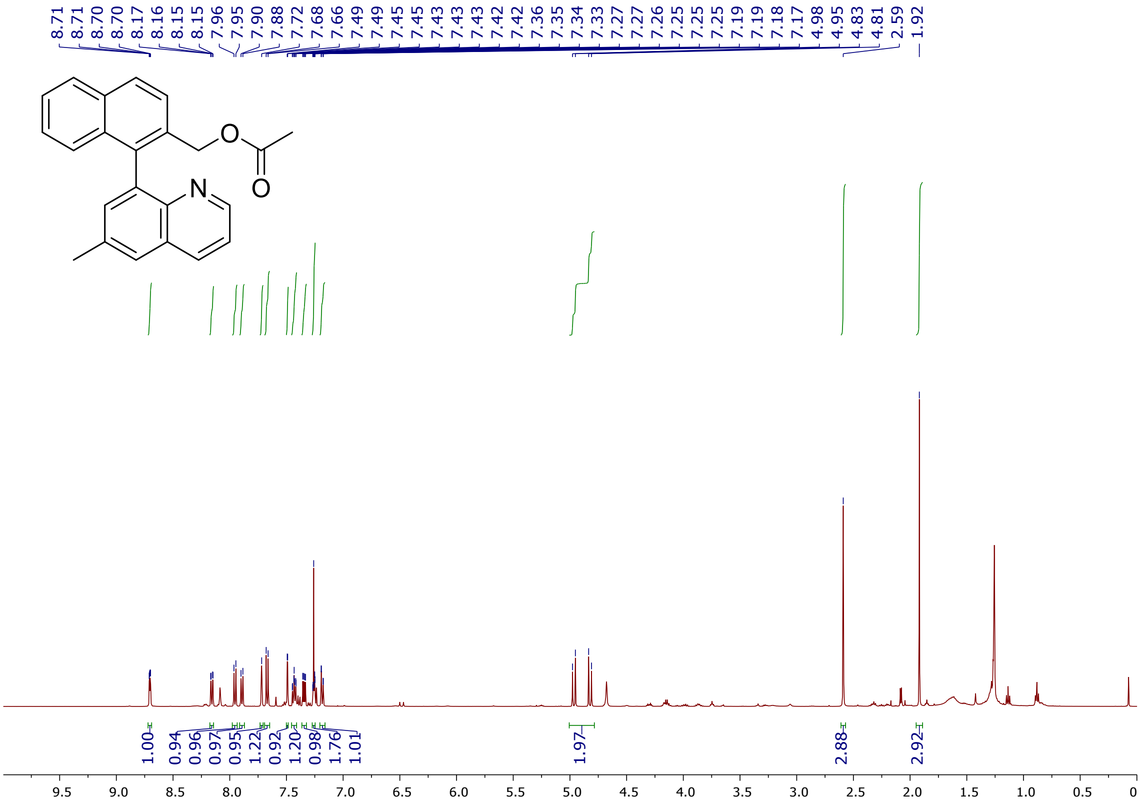

^{13}C NMR{^{1}H} (125 MHz, $CDCl_3$) of (±)-**2e**:

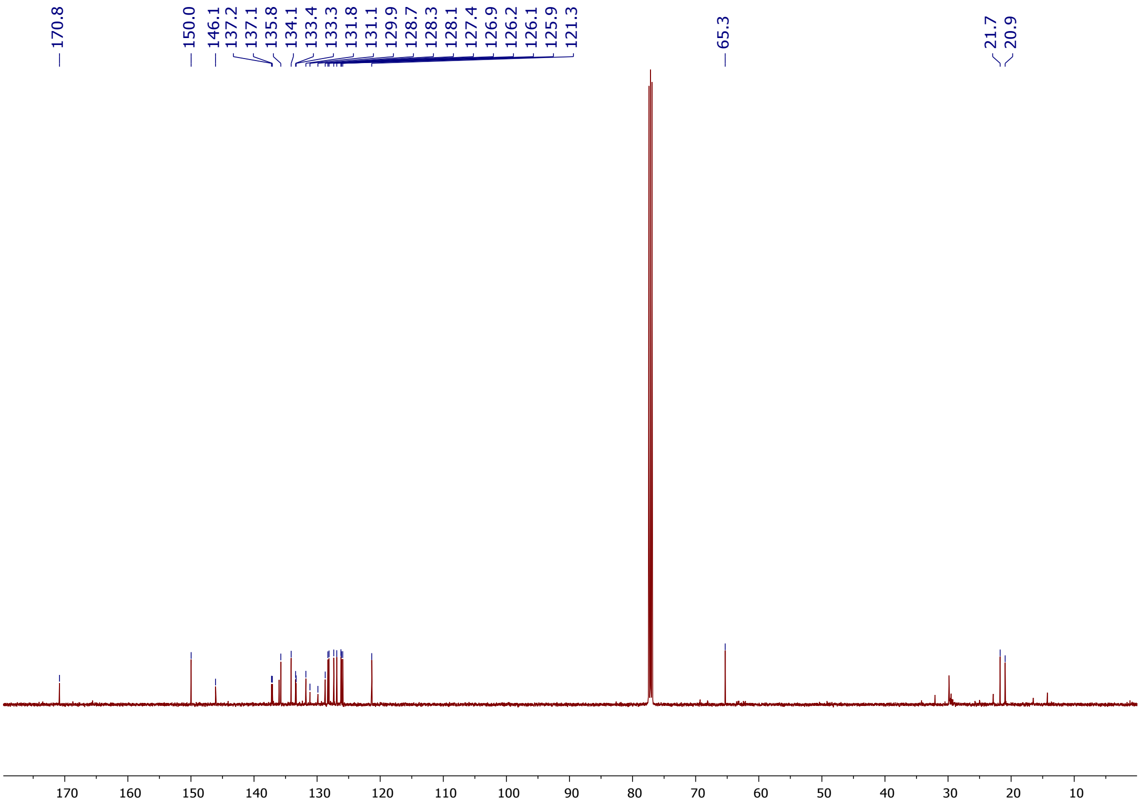

Racemic sample of **2e**: IA column, *n*-Hex/*i*-PrOH 99:1, T = 30 °C, F = 1 mL/min.

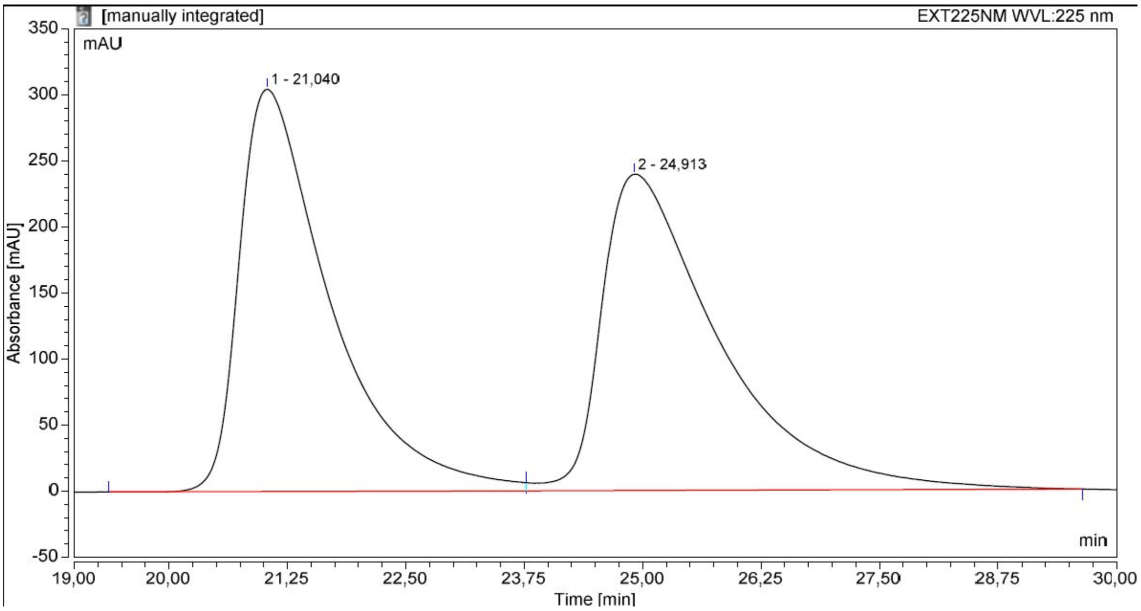

Integration Results			
No.	Retention Time min	Area mAU*min	Relative Area %
1	21,040	339,032	49,58
2	24,913	344,841	50,42

Enantioenriched sample of (*R*)-**2e**:

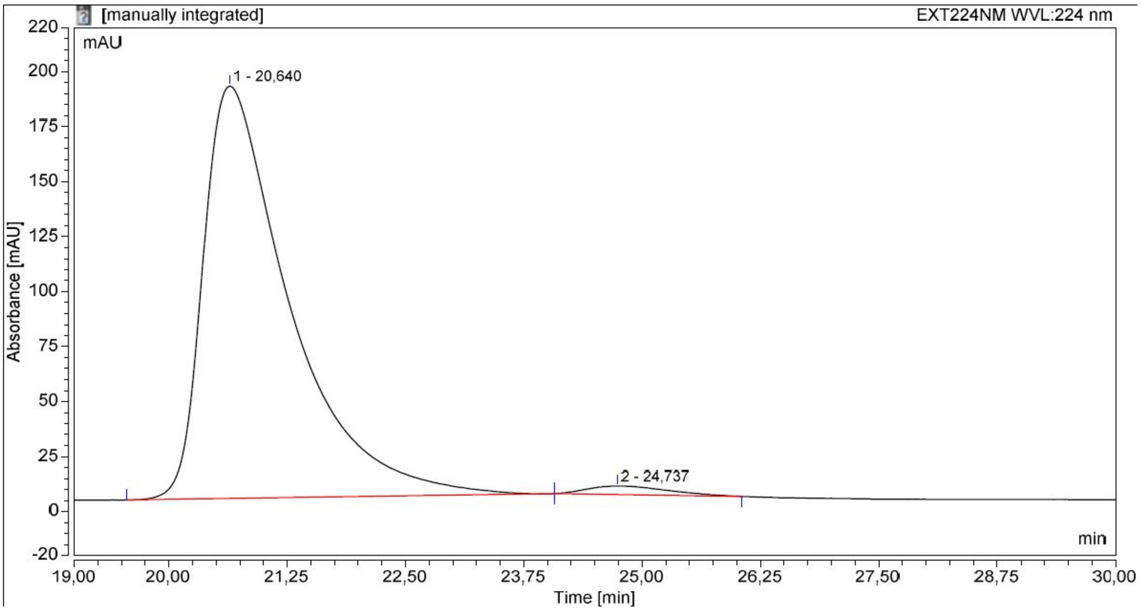

Integration Results			
No.	Retention Time min	Area mAU*min	Relative Area %
1	20,640	197,904	98,09
2	24,737	3,847	1,91

^{1}H NMR (500 MHz, $CDCl_3$) of (±)**2f**:

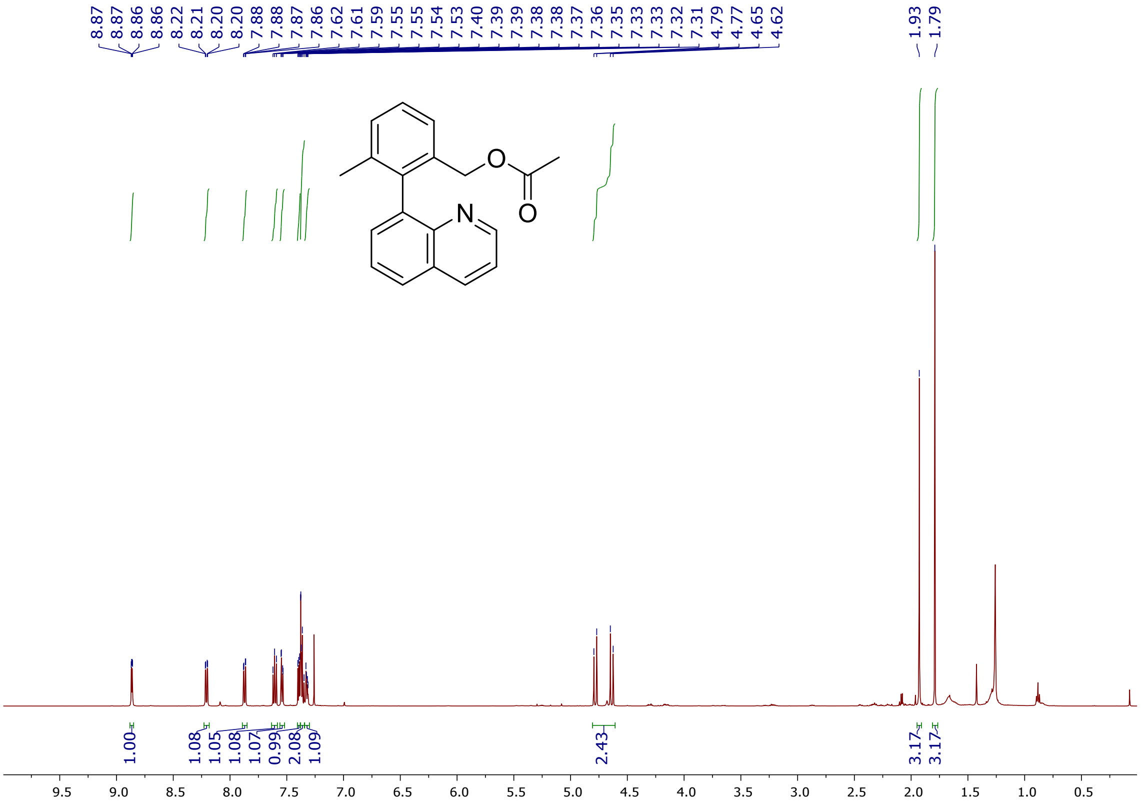

^{13}C NMR{^{1}H} (125 MHz, $CDCl_3$) of (±)-**2f**:

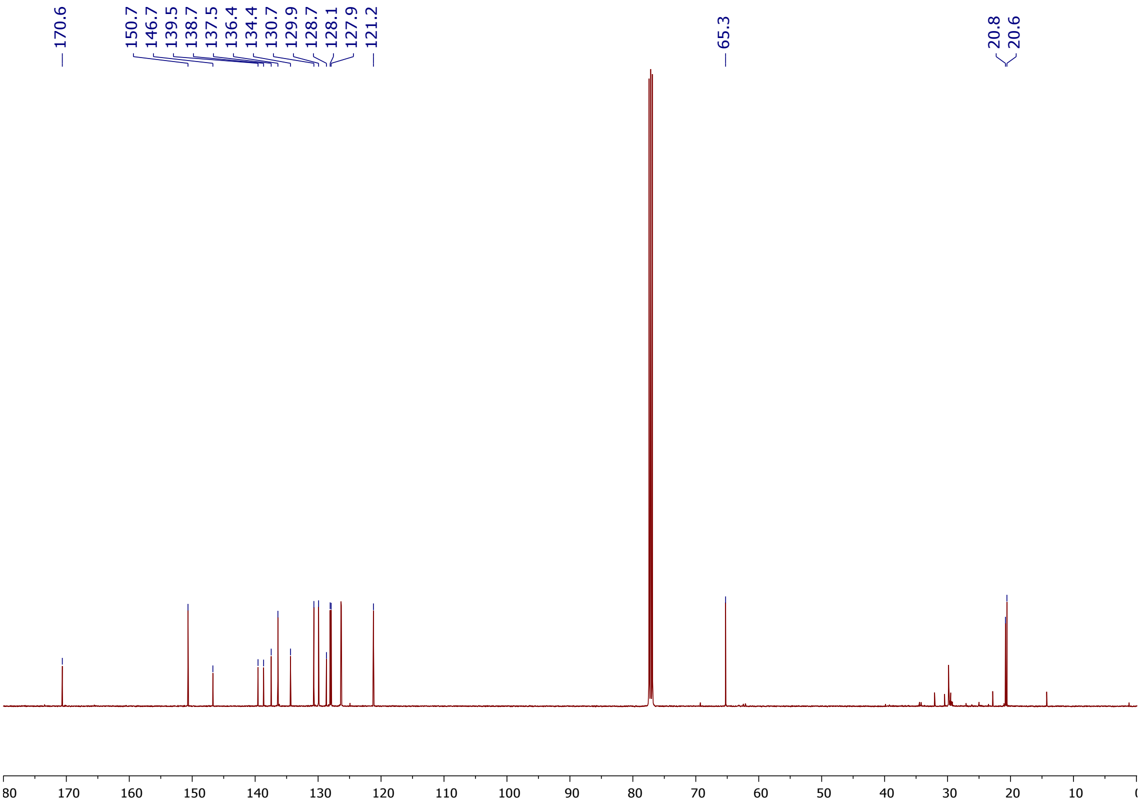

Racemic sample of **2f**: IC column, *n*-Hex/*i*-PrOH 95:5, T = 30 °C, F = 1 mL/min.

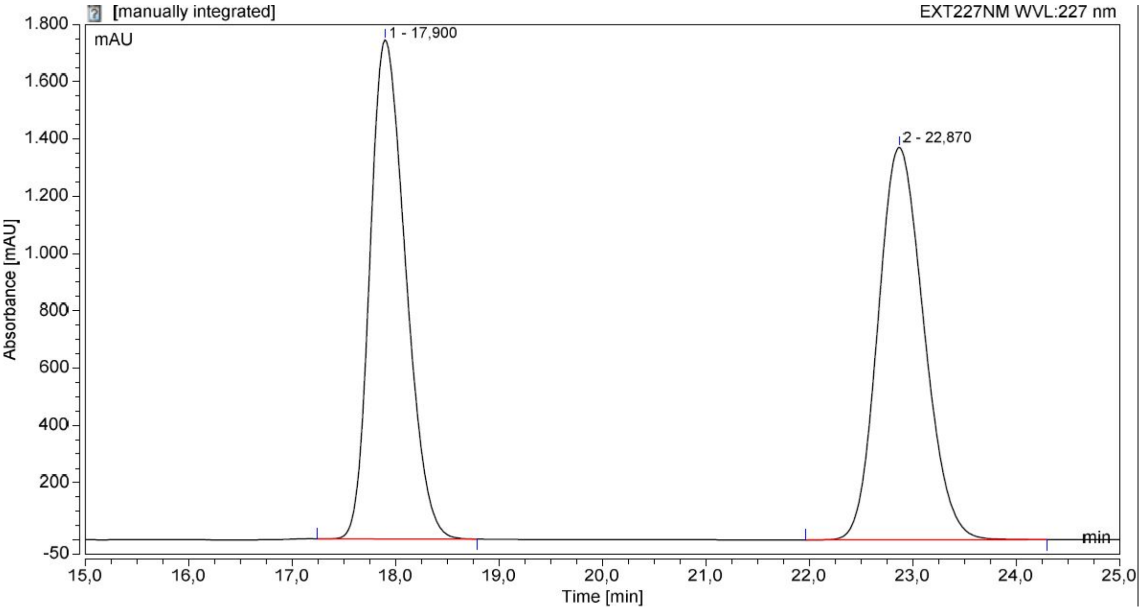

Integration Results			
No.	Retention Time min	Area mAU*min	Relative Area %
1	17,900	694,212	49,71
2	22,870	702,318	50,29

Enantioenriched sample of (*R*)-**2f**:

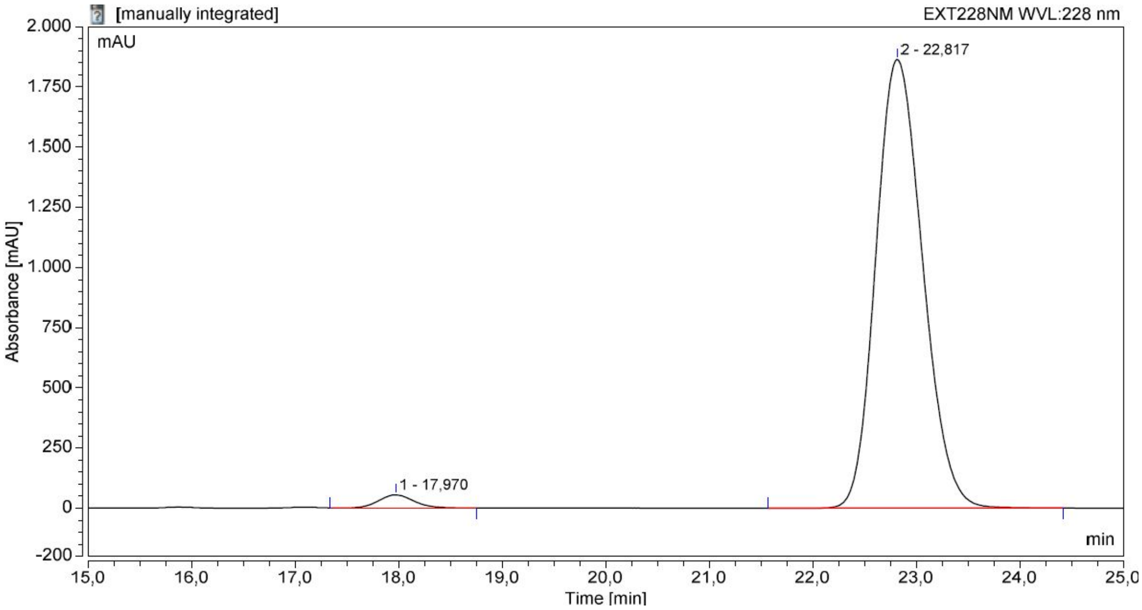

Integration Results			
No.	Retention Time min	Area mAU*min	Relative Area %
1	17,970	20,647	2,07
2	22,817	974,923	97,93

^{1}H NMR (500 MHz, $CDCl_3$) of (±)**2g**:

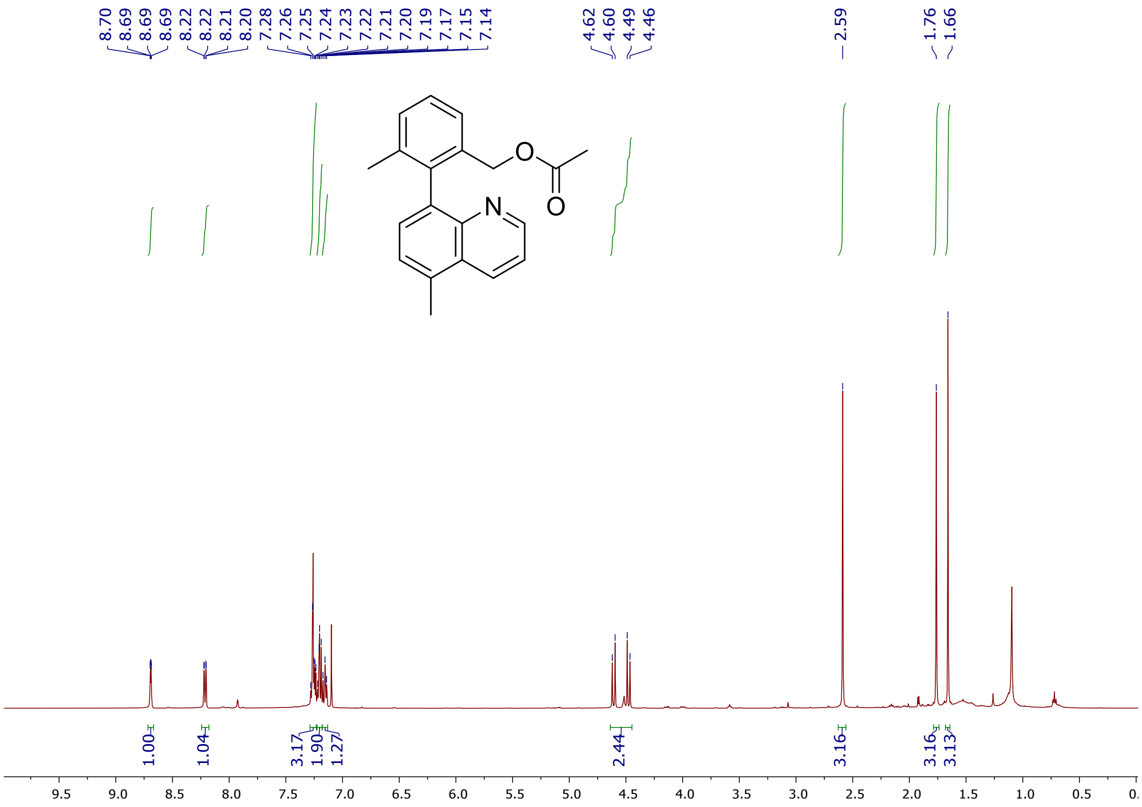

^{13}C NMR{^{1}H} (125 MHz, $CDCl_3$) of (±)-**2g**:

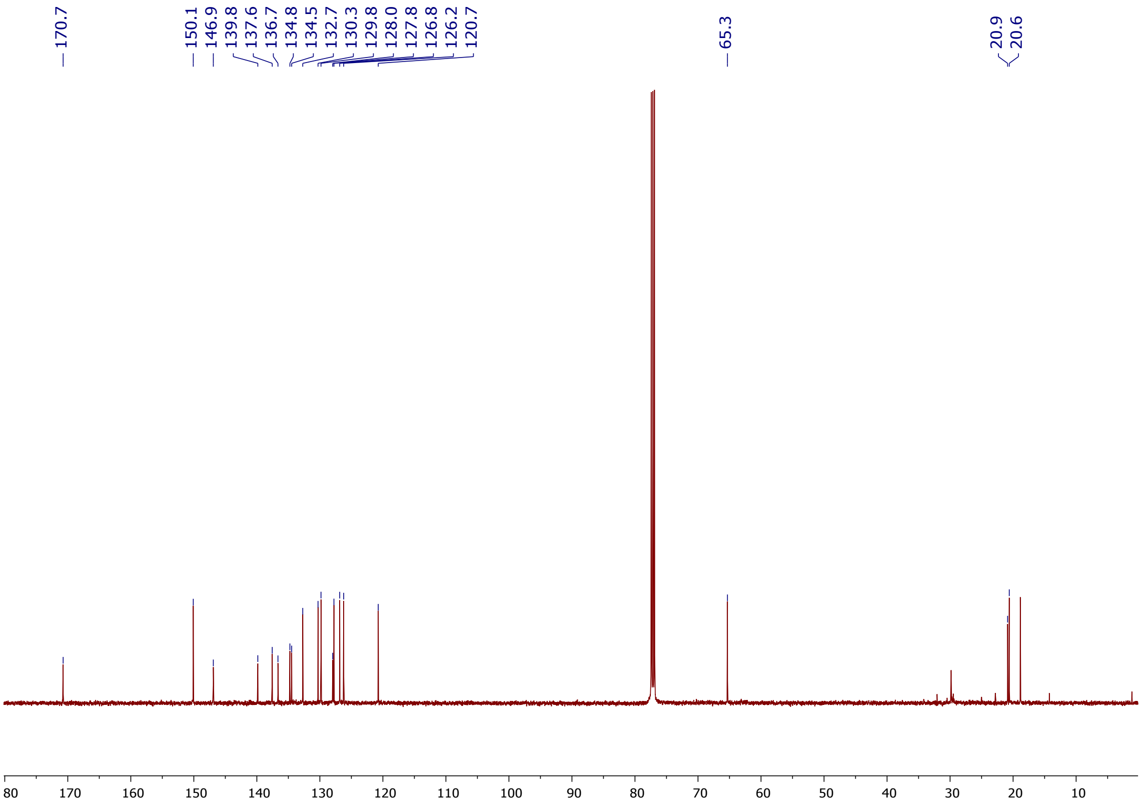

Racemic sample of **2g**: IC column, *n*-Hex/*i*-PrOH 97:3, T = 30 °C, F = 1 mL/min.

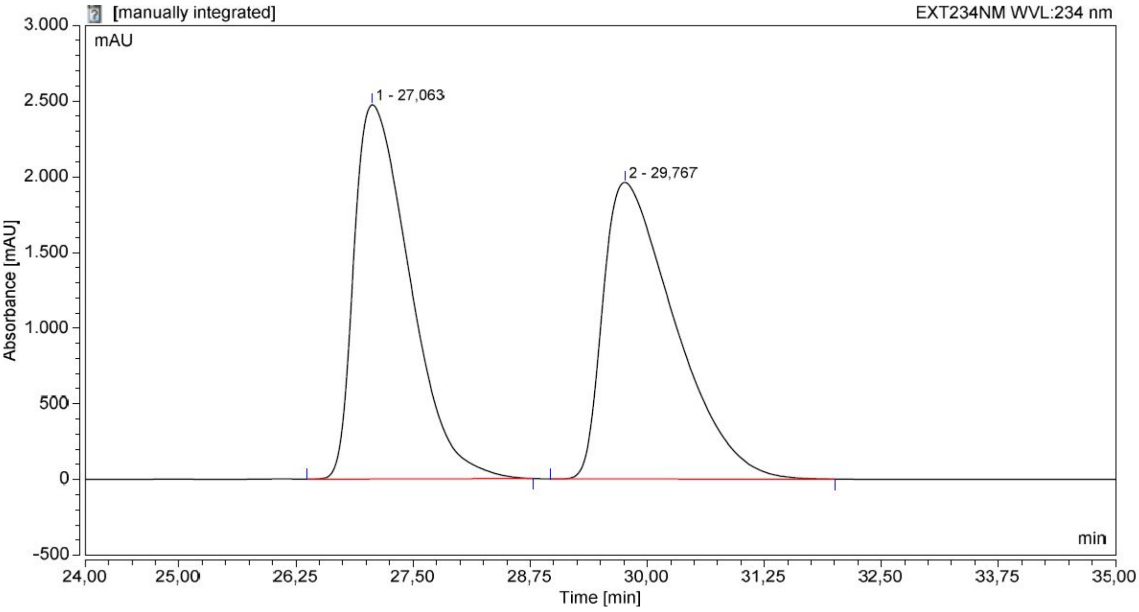

Integration Results			
No.	Retention Time<br>min	Area<br>mAU*min	Relative Area<br>%
1	27,063	1706,490	49,51
2	29,767	1740,581	50,49

Enantioenriched sample of (*R*)-**2g**:

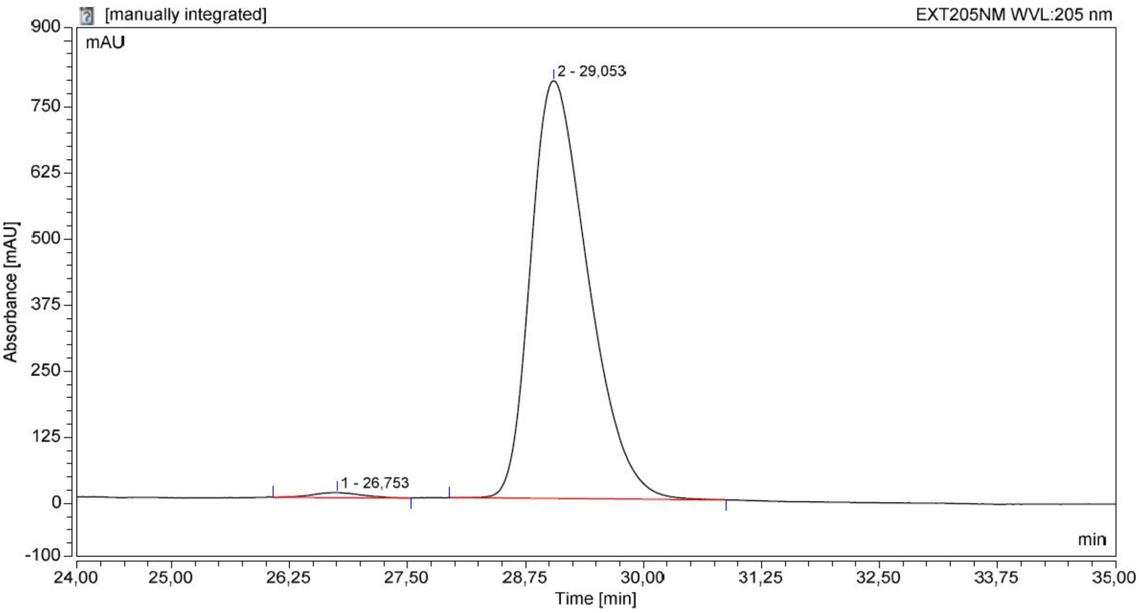

Integration Results			
No.	Retention Time<br>min	Area<br>mAU*min	Relative Area<br>%
1	26,753	5,618	1,00
2	29,053	556,586	99,00

^{1}H NMR (500 MHz, $CDCl_3$) of (±)-**2h**:

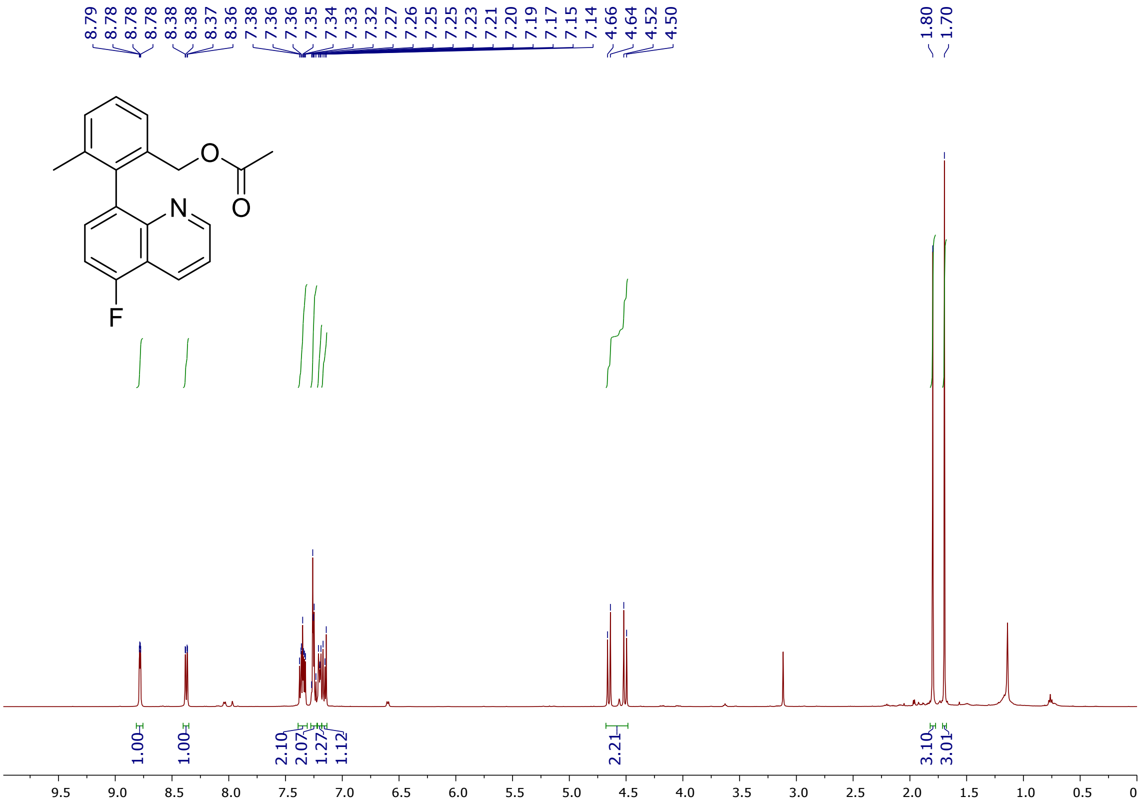

^{13}C NMR{^{1}H} (125 MHz, $CDCl_3$) of (±)-**2h**:

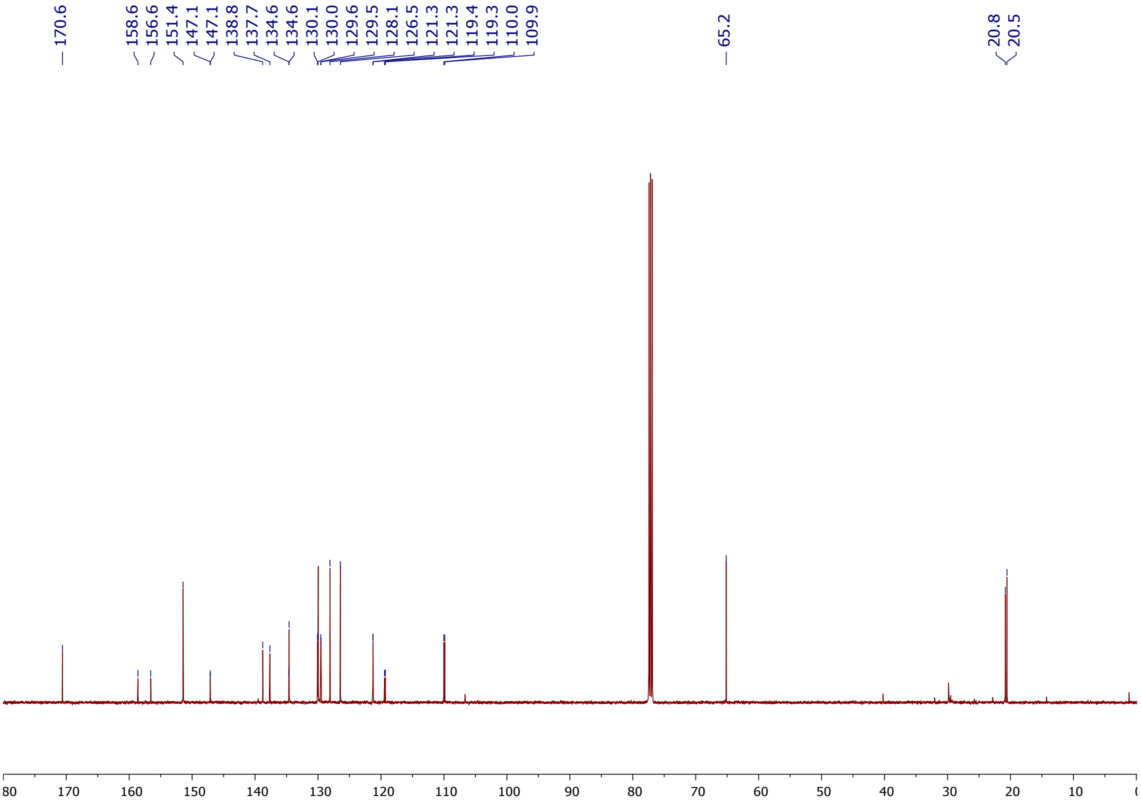

^{19}F NMR (471 MHz, $CDCl_3$) of (±)-**2h**:

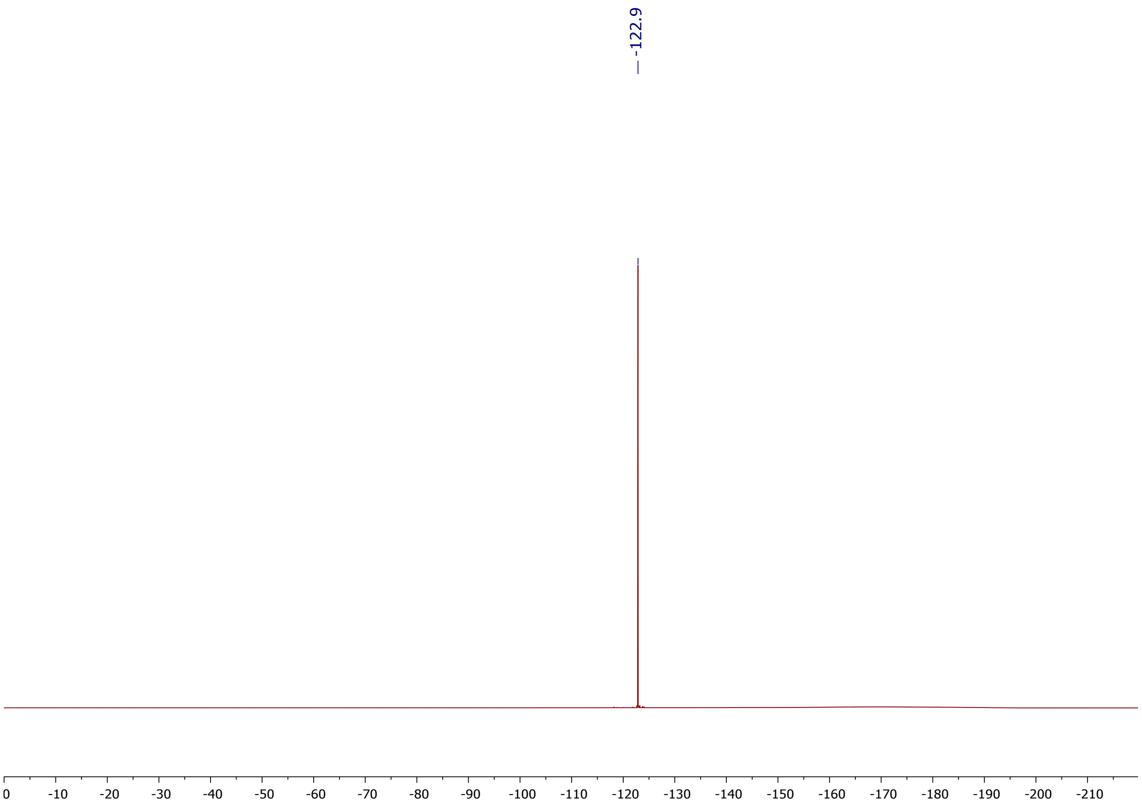

Racemic sample of (*R*)-**2h**: IC column, *n*-Hex/*i*-PrOH 99:1, T = 30 °C, F = 1 mL/min.

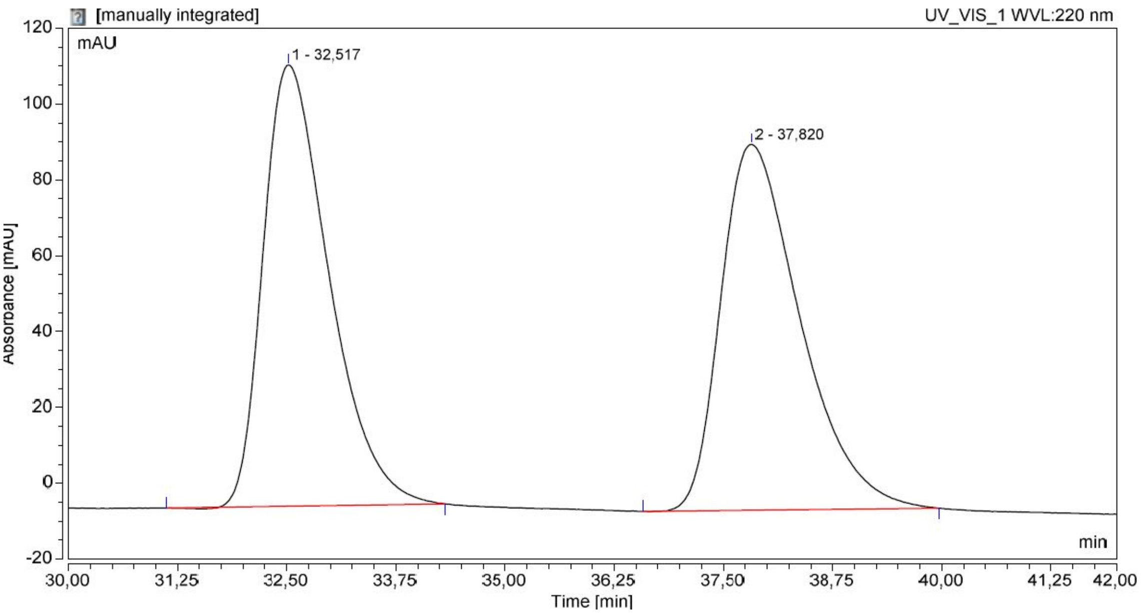

Integration Results			
No.	Retention Time min	Area mAU*min	Relative Area %
1	32,517	101,416	49,92
2	37,820	101,736	50,08

Enantioenriched sample of (*R*)-**2h**:

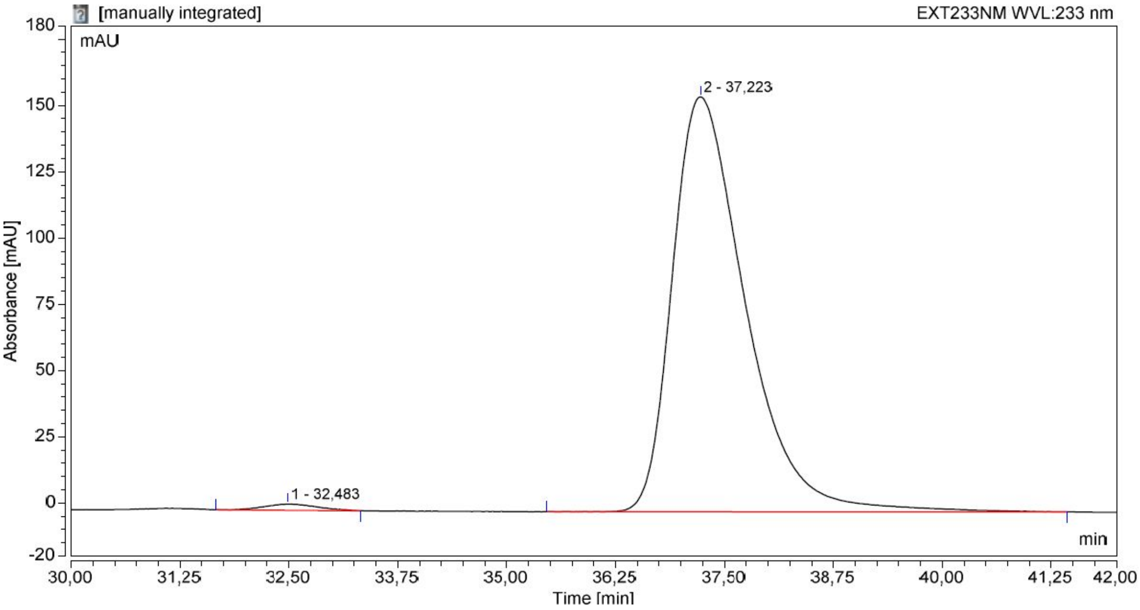

Integration Results			
No.	Retention Time min	Area mAU*min	Relative Area %
1	32,483	1,609	1,04
2	37,223	152,443	98,96

^{1}H NMR (500 MHz, $CDCl_3$) of (±)-**2i**:

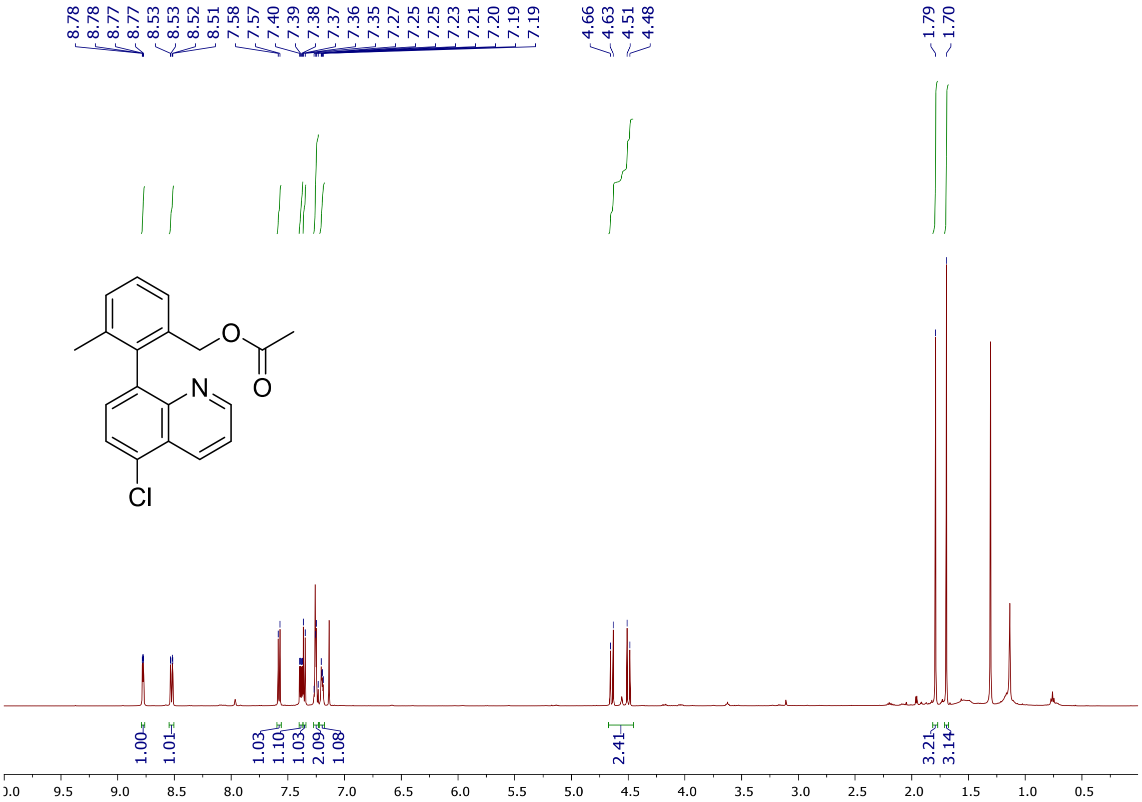

^{13}C NMR{^{1}H} (125 MHz, $CDCl_3$) of (±)-**2i**:

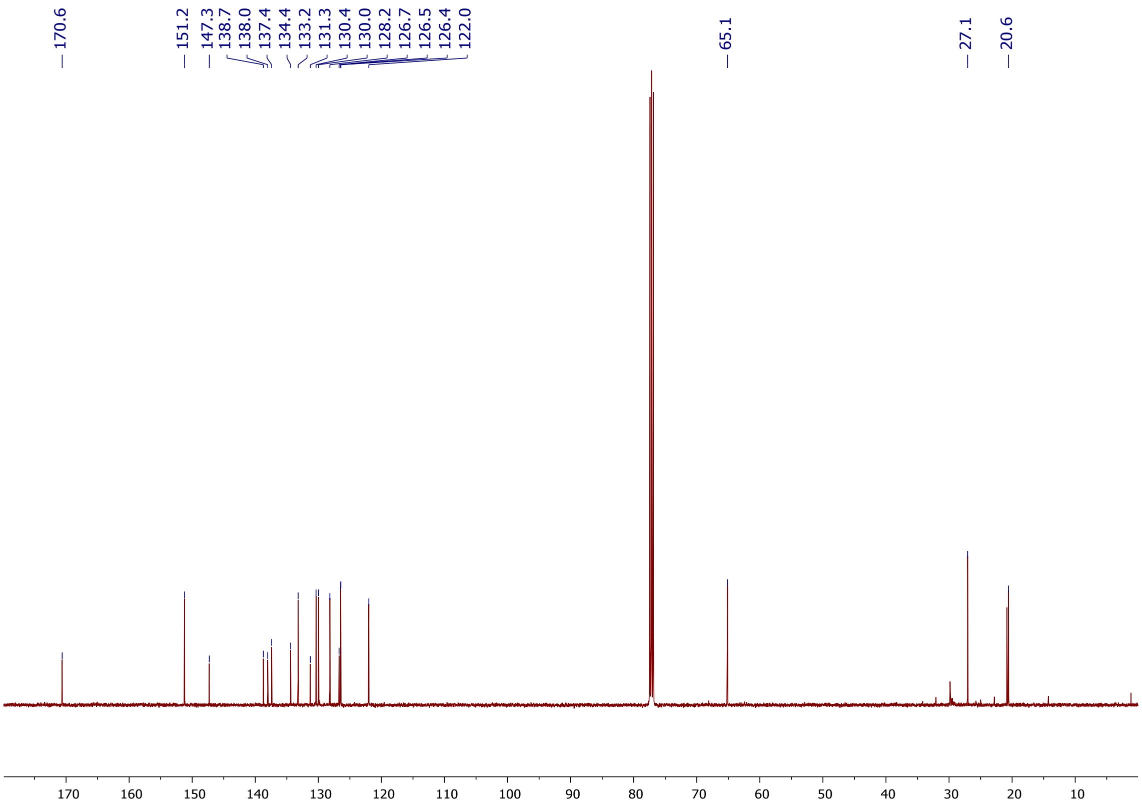

Racemic sample of (*R*)-**2i**: IC column, *n*-Hex/*i*-PrOH 99:1, T = 30 °C, F = 1 mL/min.

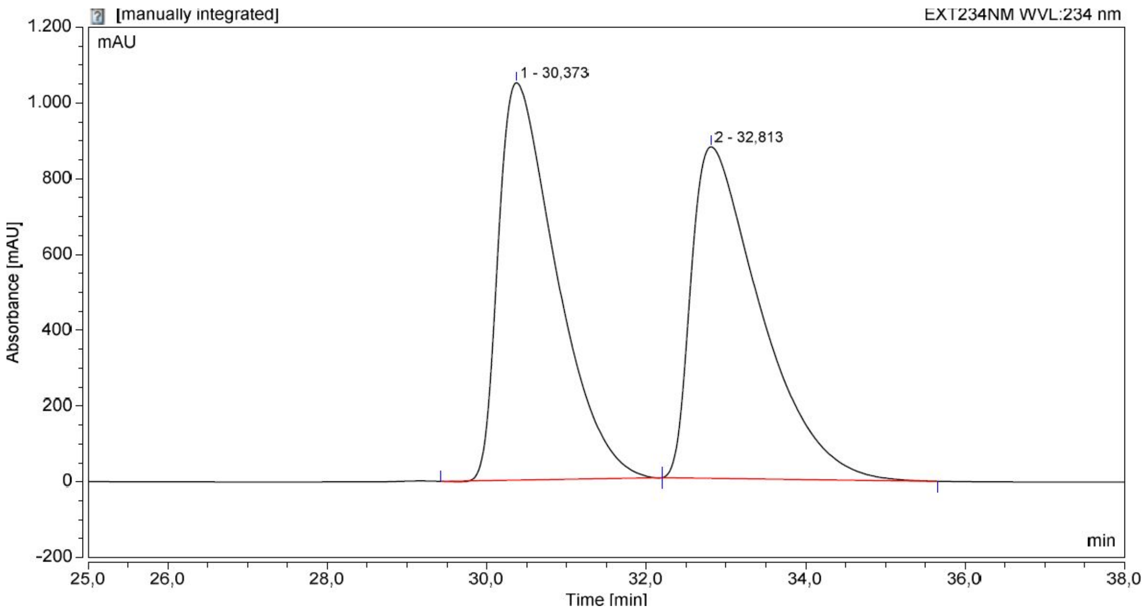

Integration Results			
No.	Retention Time<br>min	Area<br>mAU*min	Relative Area<br>%
1	30,373	881,980	49,90
2	32,813	885,525	50,10

Enantioenriched sample of (*R*)-**2i**:

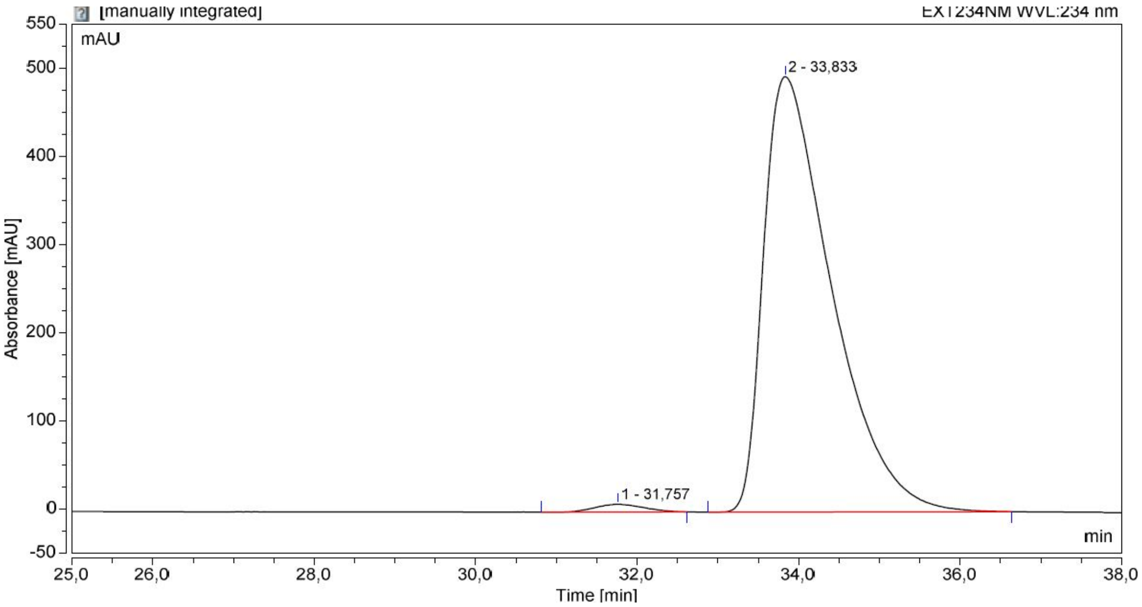

Integration Results			
No.	Retention Time<br>min	Area<br>mAU*min	Relative Area<br>%
1	31,757	6,212	1,27
2	33,833	482,434	98,73
